# Implications of climate change to the design of protected areas: The case study of small islands (Azores)

**DOI:** 10.1371/journal.pone.0218168

**Published:** 2019-06-13

**Authors:** Maria Teresa Ferreira, Pedro Cardoso, Paulo A. V. Borges, Rosalina Gabriel, Eduardo Brito de Azevedo, Rui Bento Elias

**Affiliations:** 1 CE3C –Centre for Ecology, Evolution and Environmental Changes/Azorean Biodiversity Group and Universidade dos Açores—Faculdade de Ciências Agrárias e do Ambiente, Angra do Heroísmo, Açores, Portugal; 2 LIBRe–Laboratory for Integrative Biodiversity Research, Finnish Museum of Natural History, University of Helsinki, Helsinki, Finland; 3 Group of Climate, Meteorology and Global Change of the Research Institute of Agrarian and Environmental Technologies of the University of the Azores (CMMG-IITAA), Angra do Heroísmo, Portugal; University of Nevada, Reno, UNITED STATES

## Abstract

Climate change is causing shifts in species distributions worldwide. Understanding how species distributions will change with future climate change is thus critical for conservation planning. Impacts on oceanic islands are potentially major given the disproportionate number of endemic species and the consequent risk that local extinctions might become global ones. In this study, we use species climate envelope models to evaluate the current and future potential distributions of Azorean endemic species of bryophytes, vascular plants, and arthropods on the Islands of Terceira and São Miguel in the Azores archipelago (Macaronesia). We examined projections of climate change effects on the future distributions of species with particular focus on the current protected areas. We then used spatial planning optimization software (PRION) to evaluate the effectiveness of protected areas at preserving species both in the present and future. We found that contractions of species distributions in protected areas are more likely in the largest and most populated island of São Miguel, moving from the coastal areas towards inland where the current protected areas are insufficient and inadequate to tackle species distribution shifts. There will be the need for a revision of the current protected areas in São Miguel to allow the sustainable conservation of most species, while in Terceira Island the current protected areas appear to be sufficient. Our study demonstrates the importance of these tools for informing long-term climate change adaptation planning for small islands.

## Introduction

Protected areas are created nowadays not only to maintain iconic landscapes and seascapes and ensure biodiversity conservation, but also to play a key part in the mitigation of, and/or adaptation to, climate change [1]. A common assumption is that successful conservation within protected areas is possible when these areas are managed to buffer against the processes that threaten them [2]. However, it is becoming clear that in addition to providing sustainable management of habitats and ecosystems, effective conservation strategies need to mitigate the impacts of climate change [2]. The main question is whether species range retention areas or critical areas for dispersal are covered by existing protected areas and whether there are tools to identify critical areas for biodiversity conservation in a changing climate [3]. Furthermore, the threats imposed by climate change often compound conventional threats associated with habitat degradation, pollution, poaching, and spread of alien invasive species [3]. Conservation planning is thus facing a major challenge: the need to identify climate *refugia* for conservation [4] as well as to account for new and dynamic threats emerging from climate change and their interactions with other stressors [5].

A first step towards addressing the problem of climate change in conservation planning is to project the expected impacts on biodiversity in a spatially explicit manner. Two approaches are the use of species distribution models or habitat suitability models [6–8]. These models have long been used in the context of spatial conservation planning [9, 10], and they can be used as a frame of reference for setting conservation objectives within a climate adaptation framework [11].

One of the great challenges for adaptation of species under climate change is the ability to move between protected areas as climate change may result in currently occupied areas (or locations) becoming unsuitable. [3]. However, the capacity of species to move into new areas as climate suitability shifts is likely to vary across taxa and regions. Modelling studies have shown that some species would be able to persist within existing protected areas or track climate suitability by moving into adjacent protected areas. Studies have shown that there has been a shift in distribution from groups as distinct as trees, shrubs, mammals and insects [12–14]. In some other cases, species could experience a shift in suitable habitat that is beyond their dispersal capacities [2, 15–17]. Hardships in following shifting climatic conditions are more likely for habitat and food specialist species [3] as well as for species crossing fragmented or degraded landscapes [18].

The effects of climate change on island terrestrial ecosystems are poorly known but because of their great concentrations of endemic species, islands are of disproportionate conservation importance [19–21]. Species inhabiting small islands are particularly vulnerable to extinction owing to area limitation and isolation, and the consequences of local human pressures and climate change [22, 23]. More specifically, opportunities for island species to shift their distributions along climate-relevant gradients (latitudinal, longitudinal, altitudinal) is more limited than for species in larger continental areas [24].

In order to investigate the implications of climate change to the design of protected areas we: i) analyze the sensitivity of Azorean biodiversity to projected climate changes using species distribution models (SDMs) and compare projections of species potential distributions for bryophytes, vascular plants, and arthropods between 1961–1990 and 2080–2099 (we chose these time periods because they are the standard periods recommended by the IPCC); and ii) evaluate the effectiveness with which the current protected areas of the islands of São Miguel and Terceira retain potential distributions of species under climate change scenarios and identify new priority areas for biodiversity conservation using spatial planning optimization techniques.

## Methods

### Study area

The Azorean archipelago stretches out over 615 km in the North Atlantic Ocean (37–40°N, 25–31°W), 1584 km west of mainland Portugal and 2150 km east of the North American continent. It is comprised of nine main islands of recent volcanic origin, distributed in three groups: the western group of Corvo and Flores; the central group of Faial, Pico, Graciosa, São Jorge, and Terceira; and the eastern group of São Miguel and Santa Maria. The current protected areas were established in 2007 with nine Island Nature Parks (one for each island). The criteria used to delineate the Nature Parks (Protected Areas) were the ones developed by the International Union for Conservation of Nature (IUCN) assigning different categories of protection to existing (Natura 2000, Ramsar sites, regional woodland reserves, etc.) and potential natural sites [25]. This study focuses on Terceira and São Miguel (S1 Fig), two of the largest, better studied, more populated, and economically, most important islands of the archipelago. The two islands differ greatly in the current area of protected pristine forest, with Terceira (400.3 Km^2^) having the largest area of well-preserved continuous natural vegetation in Azores (23.45Km^2^), while São Miguel (744.56 Km^2^) has only a very small area of pristine natural vegetation (3.31 Km^2^) [26]. However, São Miguel has a high proportion of single island endemics (13%) [27] for a small proportion of protected pristine forests (S1 Table).

### Species data

We analyzed bryophytes (Divisions Bryophyta and Marchantiophyta), vascular plants (Divisions Lycopodiophyta, Pteridophyta and Magnoliophyta) and arthropods (mostly from the orders Araneae, Coleoptera, Diptera, and Lepidoptera), all extensively sampled since 1980 to present over the entire archipelago. A total of 7 species of bryophytes, 50 species of vascular plants and 122 species of arthropods were used for the analysis (S1 Table). The chosen species were all the Azorean endemic species whose distribution records for the Azores had a sufficient number of occurrence records to be modelled (15 occurrences minimum, to have at least five points per climatic variable).

Records of the species presence on islands were collected using ATLANTIS 3.1 database (http://www.atlantis.angra.uac.pt/atlantis; see also http://azoresbioportal.uac.pt) [27]. The database stores detailed information about the taxonomy and the distribution of all species in the geographical areas of interest. The biological data available is at a 500 m × 500 m cell level. We assembled data from all the islands of the archipelago in order to get the largest possible range of climatic conditions where the species were shown to occur (for a more detailed description see [28]).

### Climate data

We assembled climate, topography, and geology data for the two islands into a comprehensive database. Raster layers were built at a resolution of 100 x 100 m corresponding to UTM grid-cells. Altitude, slope and aspect were derived from a digital elevation model (DEM) of 100 m spatial resolution. The DEM was developed by interpolating the values of altitude isolines from the Digital Chart (DC) provided by the Cartographic Service of the Portuguese Army. For the climatic data, we used the CIELO Model [29–31], run under the framework of the Project PROAAcXXIs (2016). The CIELO model is a simple layer model, based on the transformations experienced by an air mass crossing over a mountain, and simulates the change in the physical properties from the sea level up to the mountain. The model has been developed in a raster GIS environment and can be applied in order to get an appropriate spatial distribution of any specific climatic variable over the island. Using the capabilities of the GIS, namely integrating other different spatially distributed parameters, the model CIELO can combine several climatic variables and produce spatially detailed distribution outputs. [30]. A high number of climatic variables related to temperature, rainfall, relative humidity and solar radiation were obtained (S2 Table). To minimize collinearity among climatic variables, ordination techniques were applied to reduce the multidimensional space to a new subset of factors that characterized the main trends of environmental variation on the islands. Here we use three climatic variables: maximum annual temperature (tmax), minimum annual precipitation (ppmin), and annual range of precipitation (prange). Two time periods were modelled: 1961–1990, and 2080–2099. The choice of the baseline period of 1961–1990 is based on the recommendations of the IPCC (Intergovernmental Panel on Climate Change) [32], which endorses the use of this time period as the baseline for climate change impact studies. The CIELO model run for the future (2080–2099) was based on the Representative Concentration Pathways (RCPs) scenarios from the fifth Assessment Report [32]. We deliberately chose the “worst case scenario” RCP8.5 because it is consistent with both the historical and current trends of greenhouse gas emissions [32, 33]. The original spatial resolution of the climatic model was of 100m x 100 m and later resampled to 500m x 500m to match species data.

### Species distribution modelling

A set of species distribution models (SDMs) was generated for each species, using off-the-shelf BIOENSEMBLES software [34]. BIOENSEMBLES is a platform for projecting species distributions that includes 14 different ecological niche modelling techniques and advanced consensus projecting methodologies (S3 Table) [25]. It is a windows-based program written in Delphi that integrates with R [35] and with the Java-based Maxent [36]. Species distribution models were generated for each modelling technique, and evaluated by performing ten random splits of the species distribution data into two sets: the calibration set, with 80% of the data, used to create the models and the validation set, with the remaining 20% of the data, used to evaluate model performance [37]. This evaluation was done using True Skill Statistics (TSS) method [38], in which accuracy values vary between −1 and 1, where 1 indicates perfect agreement and values of zero or less indicate a performance no better than random. All models with TSS smaller than zero were discarded and the remaining models were evenly weighted into a consensus model of species potential distributions This ensemble model was projected for two sets of climate conditions, pertaining to two time periods: 1961–1990 (a baseline scenario) and 2080–2099 (a future scenario). In the resulting consensus maps of species potential distributions according to their climate niches, species are only considered as potentially present in a given cell if this presence is predicted by at least half of the models used.

### Optimization of conservation areas

The distribution maps for each species were overlaid using the DIVA-GIS software [39], creating maps of cumulative number of species per cell, for each taxonomic group and for each time period. The maps of the distributions, both present and future, were overlaid with the existing protected areas and the proportion of species distribution area covered by these was calculated. The mean proportion of species suitable climate space covered by current protected areas was calculated by dividing the number of cells of the suitable climate space for each species that was located inside the current protected areas by the total number of cells of species suitable climate space on the islands. We then calculated the mean of these proportions per taxonomic group, per time period. The mean loss of species suitable climate space within current protected areas was calculated as the difference in number of cells within the protected areas between the two time periods divided by the total number of cells within the protected area, per species. We then calculated the mean of the total per taxonomic group, per island.

In order to determine if the existing conservation areas are optimized to for supporting current and future species distributions, we used PRION: PRIority Optimization aNalysis v0.11 (http://biodiversityresearch.org/software/) a software developed to optimize conservation areas. This software uses a stochastic global optimization technique, which is a technique that uses mathematical optimization for selecting the best element within given criteria. In this case, the software uses genetic algorithms [40] (which are simple to implement) that use operators such as mutation, crossover and selection, to maximize the representation of features of interest (species) while minimizing the costs of each option (in the simplest case the number of cells to be protected). In this case, we used the default genetic criteria in the software, *i*. *e*. a crossover rate of 90.0 (out of 100) and a mutation rate of 50.0 (out of 100). We used an initial population size of 1000 and ran the software for 100000 generations. The software allows creation of a potential network of areas that will maximize protection of a population of a given species using the minimum area necessary (in this case number of cells). A weighted combination of representation (number of cells), cost and connectivity produces a fitness value for each solution explored by the algorithm. In this particular implementation, the analysis is spatially-explicit, in the sense that all steps of the algorithm are made directly using the spatial raster layers [41]. For this case, nearby cells have higher probabilities of belonging to the same protection class (in the simplest case either protected or unprotected). Both targets and costs may be used as restrictions, i.e., solutions that do not reach all targets or that exceed the maximum cost have low or null fitness [42].

The layers of the projected distributions for the 1961–90 and the 2080–99 periods for all the taxonomic groups together and the three taxonomic groups separately were loaded into the software. The assessment of efficiency of current Protected Areas (PAs) was performed in two different ways. The first was a typical minimum set cover problem. We compared the extent (here used as cost measure) of current PAs with the extent of a quasi-optimal solution that minimized number of protected cells while protecting the same mean proportion of each species range. This mean proportion was used as the target in the general analysis, with solutions being restricted to those that fulfilled it (hereinafter designated as minimum set). We conducted a second analysis, in which we gave more weight to single island endemics (SIEs). The ratio between the extent (number of cells protected) of the quasi-optimal solution and of current PAs reflects the efficiency of PAs, with the ratio being “1” if current PAs were as good as the best solution found by PRION and approaching “0” if the PAs occupied the entire island (*i*.*e*. a purely theoretical scenario). Because the software finds a quasi-optimal solution, if the number of current protected cells matches the ones found by the program (*i*.*e*. the ratio is 1) then the current PA’s are as effective as an optimal solution. When this is not the case, and the optimal solution can protect the same proportion of species occupying less number of cells than the current PA’s then the optimal solution is more effective (*i*.*e*. the ratio is 0). The ratio was calculated with the output values for cost from PRION for both the existing PAs and the minimum set solution. The second approach was a typical maximum coverage problem. We compared the average of the proportions of species’ protected ranges with an optimal solution that maximized species coverage with similar costs. The number of protected cells (costs) in the current PAs was used as restriction in PRION (hereinafter designated as maximum coverage). The ratio between the current and the quasi-optimal percentages of targets reached (as single output values given by PRION) reflected the efficiency of current PAs, with the ratio being “1” if current PAs were as good as the best solution found by Prion and “0” if the PAs did not reach any target.

Both the ‘minimum set’ and ‘maximum coverage’ were run for each individual taxonomic group and for the complete set of species in the study. The statistical significance of results was also assessed comparing them to the outcomes from null models in order to assess if these solutions were better than expected by chance in achieving overall and species targets. The null models consisted of simple random resampling with no further restrictions. We ran these for both time periods. For the time period of 2080–2099, we did not use the species whose distribution for this period had no projected suitable climate space.

## Results

Of the 179 analyzed species, 23 were SIE’s 13 of which are coastal species. The results from overlaying the species potential distributions with the current protected areas showed that the average coverage percentage is 53±16%, with only the Bryophytes in Terceira Island having values above 44±13%, while the remainder values average around 30% for the three taxonomic groups (Table 1).

**Table 1:** Proportion of species suitable climate space in the protected areas. Mean percentage of species suitable climate space that is covered by the existing protected areas for the three taxonomic groups for both time periods studied. The value of proportion is calculated dividing the number of cells of the projected species suitable climate space inside the existing PA’s by the total number of cells of the projected species suitable climate space.

Mean proportion of species suitable climate space covered by current protected areas (%)				
	Terceira		São Miguel	
Taxonomic group	1961–1990	2080–2099	1961–1990	2080–2099
Bryophytes	44±13	53±16	23±8	22±8
Vascular plants	36±4	38±4	29±3	34±3
Arthropods	27±3	33±3	23±1	26±2

Note: The projected climate space for most species in all taxonomic groups is reduced from 1961–1990 to 2080–2099 [28], and so even though the numbers increase from one time period to the other this is just because there is an overall decrease in species potential distribution.

**Fig 1:**
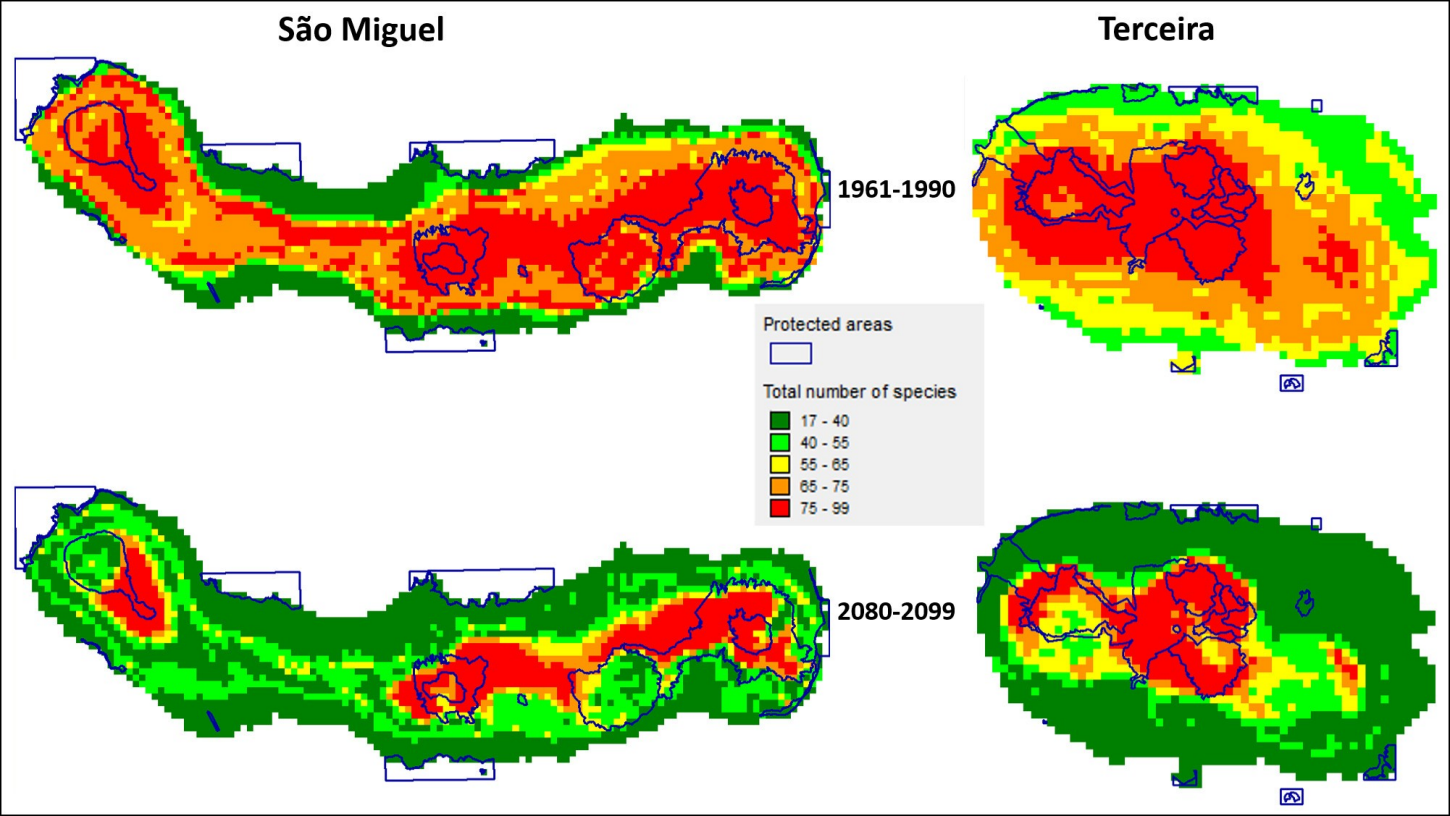
Maps of cumulative number of species for both Islands. Current Protected areas and total number of species per cell for the three taxonomic groups in São Miguel and Terceira Islands for the 1961–1990 and 2080–2099 time periods.

Considering the potential distribution of species only within the boundaries of the current PAs, there is a loss from one period to the other. The mean loss of potential distribution within the protected areas in the future projections in vascular plants is as much as 37±4% while the arthropods will have a 50±3% loss (Table 2). There is a reduction in the overall distribution of the species from one time period to the other, although for Terceira the current protected areas still contain much of the diversity within its boundaries (Fig 1).

**Table 2:** Mean loss of suitable climate space within protected areas for the three taxonomic groups for both time periods. The loss of species suitable climate space within the current PA’s is calculated as the difference between the number of cells of species suitable climate space for the 2080–99 period and the 1961–90 period over the total number of cells of the PA.

Mean loss of species suitable climate space within current protected areas (%)		
Taxonomic group	Terceira	São Miguel
Bryophytes	48±18	33±15
Vascular plants	33±5	37±4
Arthropods	45±4	50±3

The results from the PRION analysis show how the projected distributions of all the taxonomic groups combined vs separate yield different results, among groups and between islands (Table 3). The effectiveness of the current PAs is higher in Terceira Island, with the lowest value of efficiency (0.641) found for the Bryophytes Minimum Set 2080–99 projections. For São Miguel the effectiveness of the current protected areas is much lower than Terceira, with values reaching 0.405 (when not considering the SIE’s).

**Table 3:** Efficiency of the current protected areas considering the projected distributions of all three taxonomic groups. The taxonomic groups were considered together and separately using the Minimum set and Maximum coverage scenarios for both the 1961–90 and 2080–99 time periods. Minimum set values are a ratio between PRION output values for costs for the quasi-optimal solution and the current PA. Maximum coverage values are a ratio between the PRION output values for the targets of the quasi-optimal solution and the current PA. Efficiency is measured as the ratio between the existing PA’s and quasi-optimal solutions found by the software. The closer to 1 the values are, the more efficient the current PA’s are, as compared to the quasi-optimal solution found by the software.

	Approaches	Time period	Terceira’s Protected area efficiency	São Miguel’s Protected area efficiency
All taxonomic groups	Minimum Set	1961–90	0.907	0.735
		2080–99	0.773	0.554
	Maximum Coverage	1961–90	0.909	0.785
		2080–99	0.805	0.671
All taxonomic groups (SIE)	Minimum Set	1961–90	0.255	0.178
		2080–99	0.271	0.177
	Maximum coverage	1961–90	0.312	0.248
		2080–99	0.358	0.324
Bryophytes	Minimum Set	1961–90	0.788	0.519
		2080–99	0.641	0.405
	Maximum Coverage	1961–90	0.837	0.695
		2080–99	0.764	0.671
Vascular Plants	Minimum Set	1961–90	0.872	0.591
		2080–99	0.780	0.456
	Maximum Coverage	1961–90	0.887	0.675
		2080–99	0.791	0.589
Arthropods	Minimum Set	1961–90	0.925	0.820
		2080–99	0.746	0.630
	Maximum Coverage	1961–90	0.932	0.819
		2080–99	0.792	0.684

**Fig 2:**
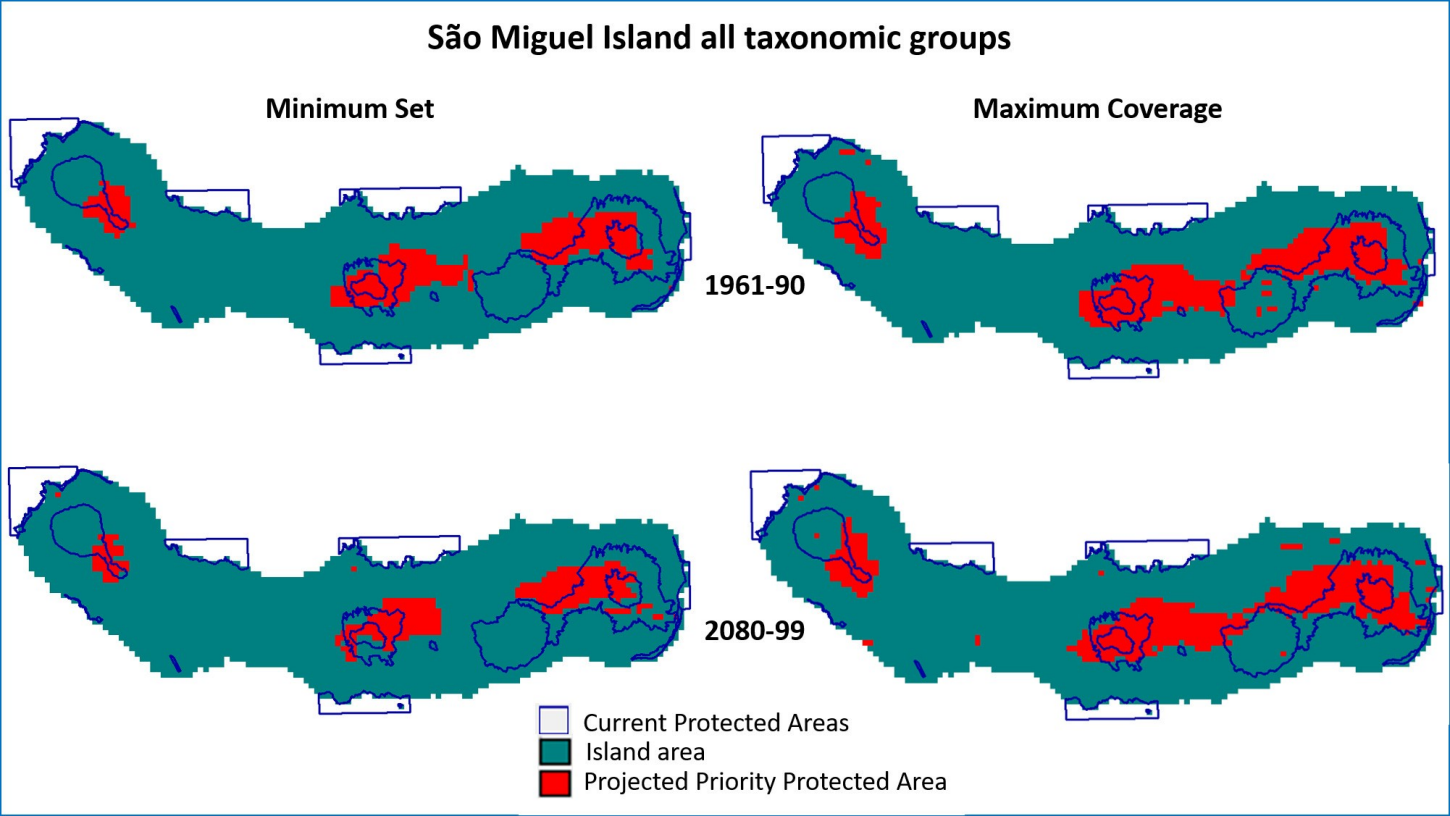
Projections for locations of Protected Areas calculated with the PRION software for São Miguel Island for the 1961–90 and 2080–99 periods for All Taxonomic groups, and the current protected areas. Minimum Set—quasi-optimal solution that minimized the protected number of cells while protecting the same average proportion of each species’ range. Maximum Coverage—the optimal solution that maximized species coverage with similar costs.

**Fig 3:**
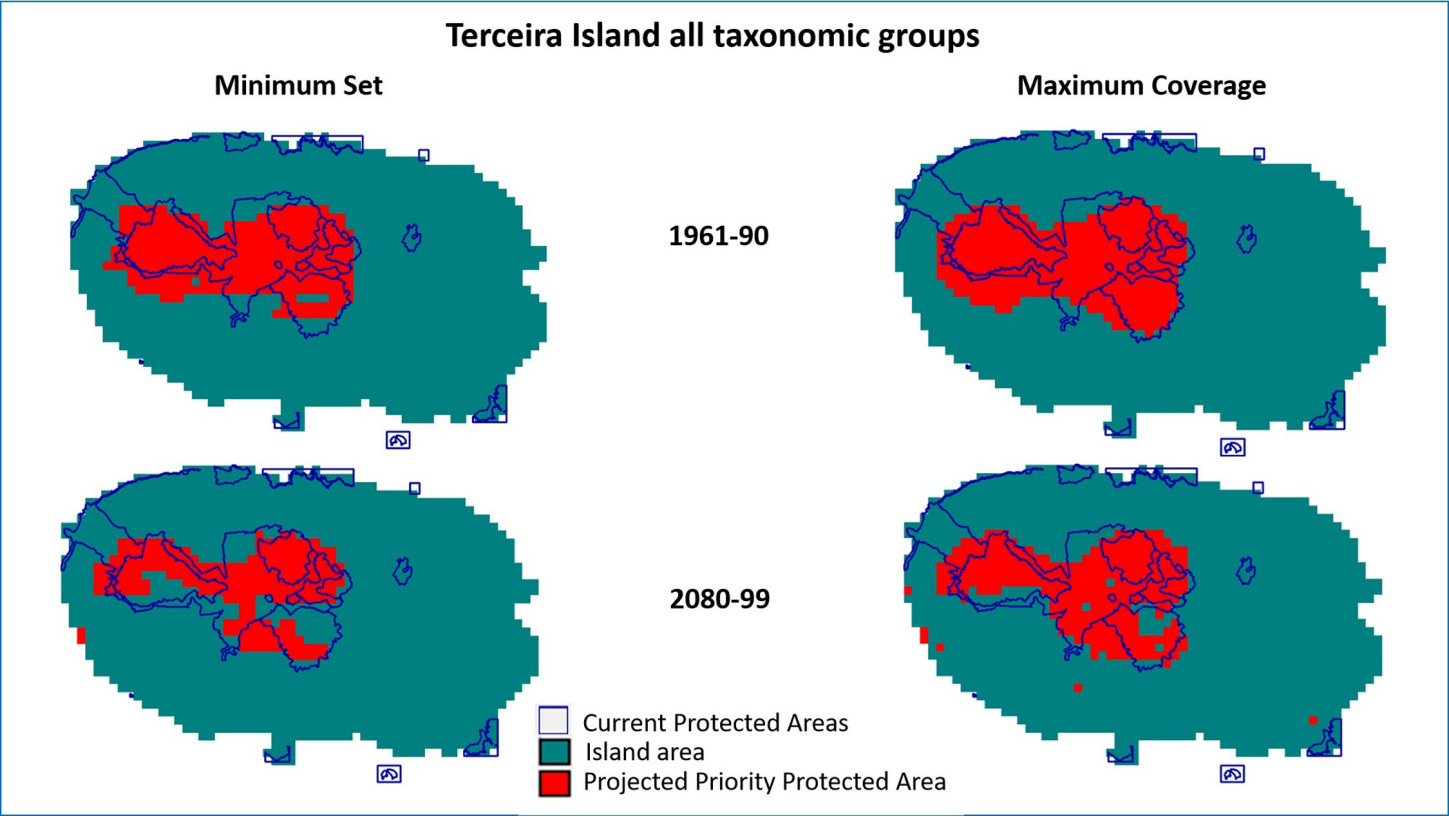
Projections for locations of protected areas calculated with the PRION software for Terceira Island for the 1961–90 and 2080–99 periods for All taxonomic groups, and the current protected areas. Minimum Set—quasi-optimal solution that minimized the protected number of cells while protecting the same average proportion of each species’ range. Maximum Coverage—the optimal solution that maximized species coverage with similar costs.

**Fig 4:**
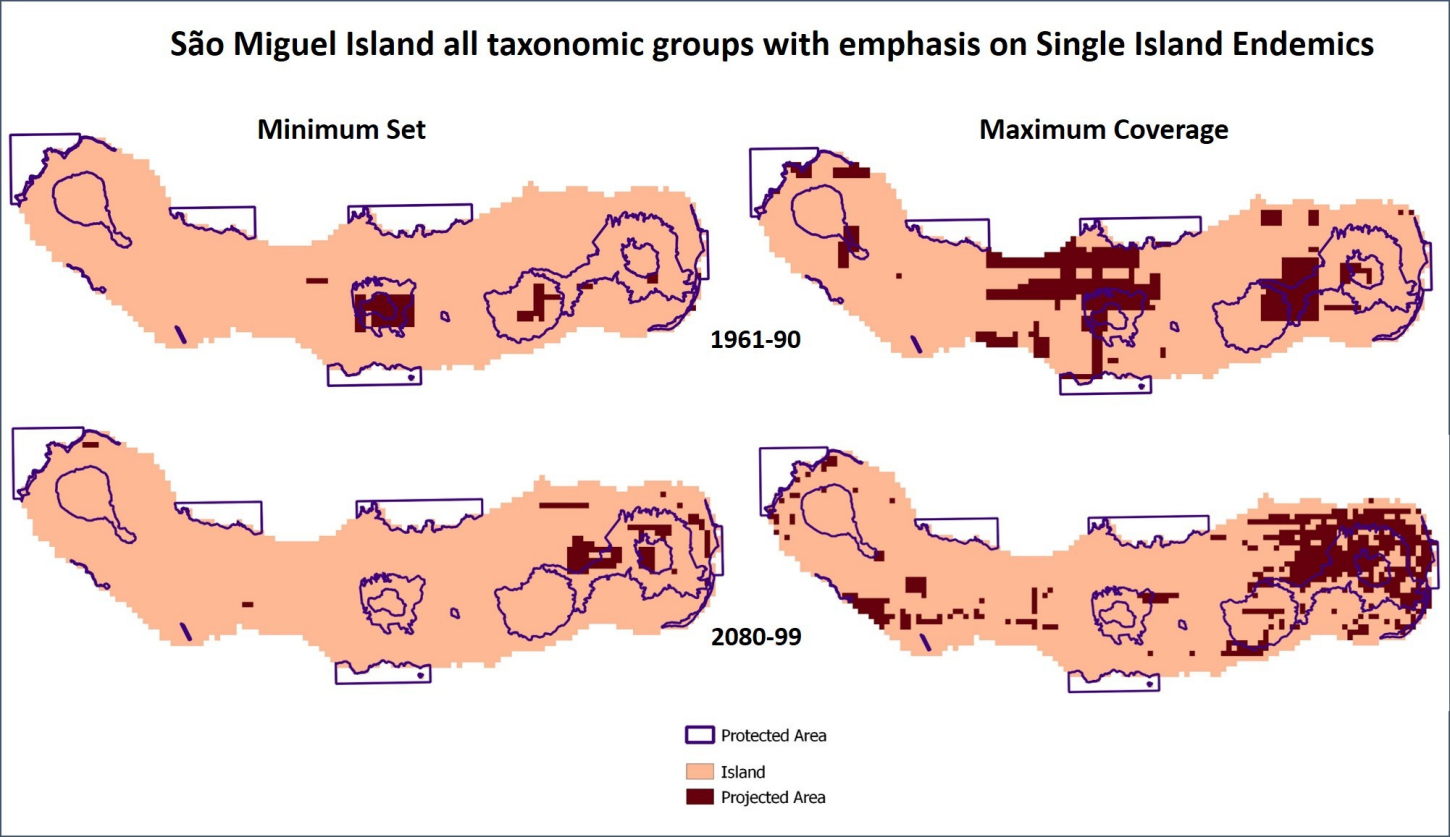
Projections for locations of protected areas calculated with the PRION software for São Miguel Island for the 1961–90 and 2080–99 periods for All Taxonomic groups with emphasis on the SIE species, and the current protected areas. Minimum Set—quasi-optimal solution that minimized the protected number of cells while protecting the same average proportion of each species’ range. Maximum Coverage—the optimal solution that maximized species coverage with similar costs.

**Fig 5:**
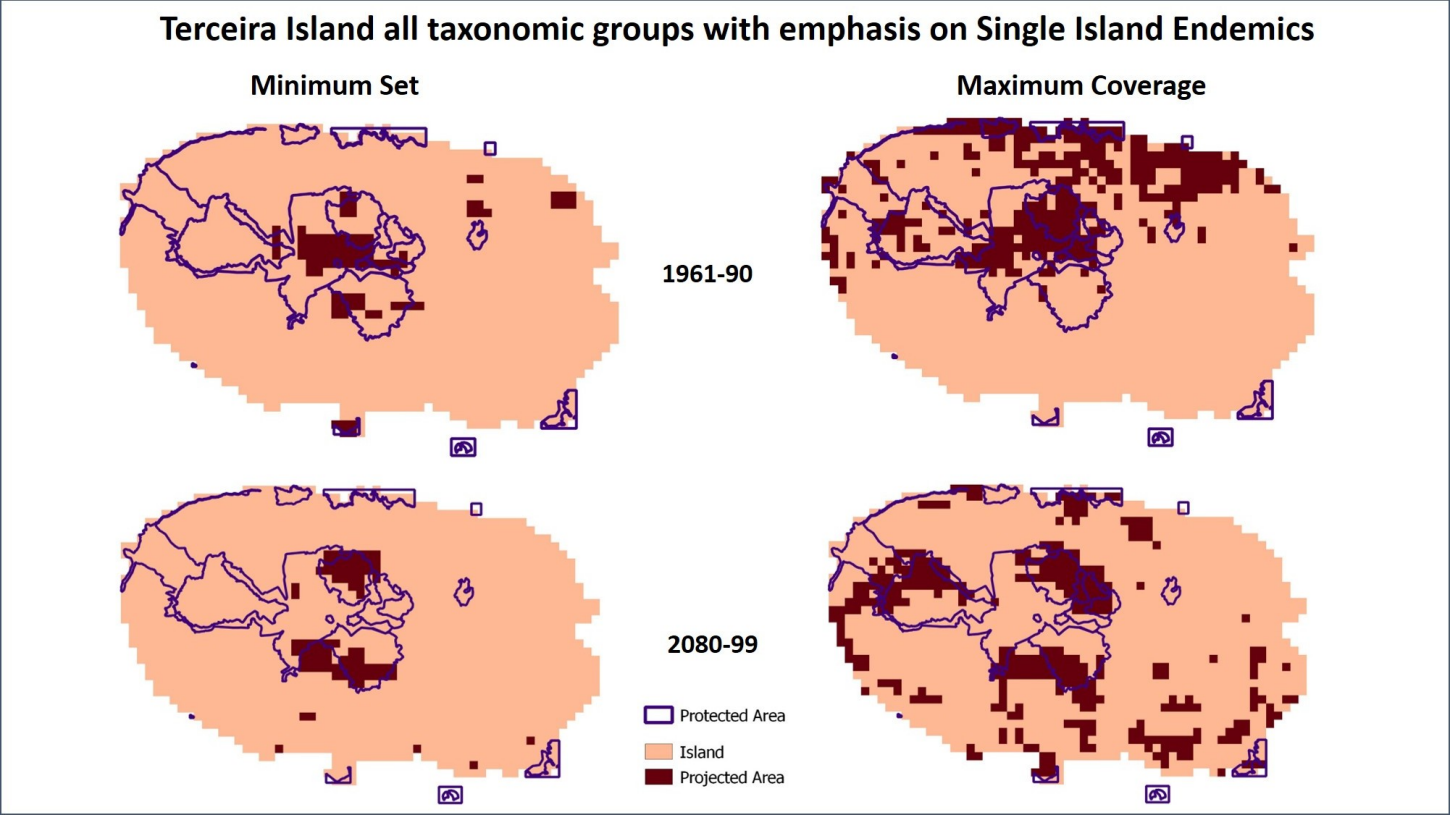
Projections for locations of protected areas calculated with the PRION software for Terceira Island for the 1961–90 and 2080–99 periods for All taxonomic groups with emphasis on SIE species, and the current protected areas. Minimum Set—quasi-optimal solution that minimized the protected number of cells while protecting the same average proportion of each species’ range. Maximum Coverage—the optimal solution that maximized species coverage with similar costs.

For São Miguel Island the PRION projections for all groups extend the projected protected areas beyond the current protected areas (Figs 2 and 3), while for Terceira this is not the case. The bryophytes and vascular plants have a similar pattern for São Miguel Island showing similar values of current PAs’ effectiveness (Table 3) (S2 and S3 Figs). Arthropods show a different pattern (S4 Fig) where the PAs efficiency is higher for both islands but more so in Terceira than in São Miguel. For the projection maps from PRION the obtained areas for the minimum set are smaller than the areas obtained by the maximum coverage (Figs 2 and 3) (S2–S7 Figs). This difference is more evident in the projections for the period 2080–99, where the loss of potential distribution range of the species seems to require less protected area in a minimum set scenario. The analysis where more weight was given to the Single Island Endemics (Table 3) show a similar pattern for the efficiency, but with much lower values. The projected areas are also located outside the existing PA’s, but with a more scattered pattern (Figs 4 and 5). Nevertheless, the results for the performance of the protected areas (current and projected) for each species showed that these solutions were all better than a random set of protected cells (S4 Table).

## Discussion

The proportion of species climate potential distribution inside the current PA’s was found to increase from the 1961–1990 period to the 2080–2099 period (Table 1), especially for Terceira. This happens because the potential distribution of the species actually decreases from one time period to the other, and proportionately the percentage of the species distribution that is covered by the current PA’s increases. However, when we compare the species potential distribution only inside the PA’s (Table 2), there is actually a decrease by over 33±5% for Terceira Island for example. This shows how important it is to have all the information possible in order to make well-founded recommendations when it comes to PAs.

There are many species whose projected distribution for the 1961–1990 period are outside the protected areas, even if the number of species per cell is higher inside these compared with non-protected areas. However, with the impacts of climate change we project a decrease in number of species per cell inside the current protected areas considering all the species together (Fig 1). From the analysis, we saw that on average less than 53±16% of the species distributions are covered by the current protected areas. This means that for most of the species their range of distribution is outside protected areas, which can increase extinction risk due to human infrastructure and associated stressors [18, 43, 44]. What we see from the values on the right of Table 1 and from Fig 1 is that the current PAs (S1 Fig) do not cover most of the species projected distribution under climate change. This type of information is important because species distribution models can be used as a frame of reference for setting objectives of conservation within an adaptive framework (a basic system of measures that can be adapted as needed)[11].

We were able to ascertain through PRION that the current PAs are still a better solution than just a random spread of protected cells, as would be expected (S4 Table). However, because most PAs have fixed boundaries, they will not protect the populations of the species whose distributions move in response to climate change [22]. Some modelling studies have shown species to move out of reserves due to climate change and as a result may require additional protected areas to achieve conservation in the future [2, 15, 16]. This is where a software like PRION can become useful.

The PRION software offers the chance to not only evaluate the effectiveness of current PAs, but it also allows for a projection of optimized areas considering the future distribution of species, allowing for a more effective response to climate change. Even though the existing PAs are projected to yield the highest number of species within the islands from all the three taxonomic groups in the 2080–99 period, when compared with the scenarios of Minimum Set or Maximum Coverage these are less effective. This shows that there are spots where the species may be protected that have not been considered and that may represent *refugia* in the future under climate change conditions. The concept of *refugia* (or *microrefugia*) has been extensively studied looking into past events [45]. However when looking into the future, seldom do studies look into the climatic change at a local scale or consider the topographic variations that can affect the possible *refugia* [45]. In this study, these variables have been taken into account. These projections show that these areas that can be possible future *refugia* may be important for the future PA management. Considering the two scenarios tested for the future distributions we can see that current PAs may be expanded. Indeed, there is a need to further evaluate where these could be placed in order to ensure that the maximum protection possible will be given in the future. Shifts in distribution of hotspots for rare species due to climate change under different future scenarios have been shown for beetles such that existing PAs system were inadequate for assuring the conservation of these species [46].

Additional analyses are necessary, however, in order to maximize protection of important endemic species within PAs, particularly SIE species. In this case we found that when more weight is given to SIEs (Figs 4 and 5) the area necessary to protect them is greatly reduced, since their projected distribution for the future scenarios is very limited, but these areas are still located primarily outside the current PA’s. Not knowing what species contribute to what ecosystem services means that the full consequences of species extinctions are extremely hard to predict [47].

Another fact to take into account is dispersal, which is one of the largest sources of uncertainty in the context of climate change conservation [48]. For example, fragmentation can limit the ability of species to move to climatically suitable areas and reduce gene flow [22]. Beyond fragmentation, loss of habitat may also hinder the movement of species (where they can move) and their ability to cope with climate change by limiting their ability to search for more suitable climatic conditions [6, 49, 50]. For São Miguel Island the maximum coverage scenario showed that a connection between the current PAs could optimize them for the future (Fig 2) (S2–S4 Figs). Moreover, in a recent study modelling several endemic insect species [51], Aparício and colleagues projected additional reduction in areas important for functional connectivity for populations of these species among current PAs in Terceira.

The results from our work show that protected areas may need more frequent revision, especially for cases like islands, where climate change is predicted to have a strong negative effect on biodiversity loss, as is the case of the Azores [28] and for all Macaronesian islands in the case of the bryophytes [52]. Because island space is limited, competition with anthropogenic uses is a potential generator of conflict. Studies have shown that a dynamic strategy for the creation of protected areas can be more effective than just adding protected areas to the existing ones under a climate change scenario [53]. The solution can be to release some of these areas where they may no longer be effective in the future in order to add different areas and so this may be more appealing to the decision makers [54]. Recently a study found that public and private protection led to different patterns of positive employment impacts indicating the importance of investing in both types of land protection to increase local opportunities. In addition, they found that the greatest magnitude of employment impacts were due to protection in more rural areas, where opportunities for both visitation and amenity-related economic growth may be greatest [55]. When we look at land use maps for the protected areas that are projected by the PRION (S8 and S9 Figs), we see that these are mainly planted forests and pastures. These areas can easily receive a level of protection (Category V of IUCN- Protected Landscape) that does not interfere with their use. Such protection can maintain important landscapes, and associated nature conservation along with other values created through traditional management practices. This could be the case for pasture land that maintains much of the native diversity. Studies have found that semi-natural pastures and exotic forests in the Azores seem to play an important role as corridors between natural forests for both endemic and native species [43]. For the case of the Azores, the regional government has a cautionary principle when creating protected areas (article 4, number 1 of the law decree DLR n.° 15/2012/A). It is the regional and local government right and obligation to ensure that there is an effective protection of the landscape. The recommendations for creating or changing protected areas is the responsibility of the environmental directorate with the help of experts that monitor and survey the different protected areas in the Azores. New boundaries or changes to the protected areas can be proposed (based on expert advice, and now using tools like PRION) and public consultation should be performed before any changes are taken into effect. Although the process to change or revise the current protected areas can be challenging it is important to allow the information on best practices to reach not only the decision makers but also the public. The use of different tools can provide useful information to the decision makers e.g., the use of remote sensing to delineate possible *refugia* areas [56]. With tools like PRION projected maps can be used to demonstrate which areas may need to be prioritized.

## Conclusion

In this work, our model projections for most endemic species studied in Terceira and São Miguel Islands suggests that there will be a decrease in distribution with climate change. This has implications for the conservation of these species, being at risk of either disappearing or reducing their distribution dramatically. Evaluating the suitability of the current protected areas has shown that these may not meet the needs of species in the future, especially on São Miguel. This type of analysis can be done for other islands of the Azores as well. We have shown that tools like the software PRION useful for delineating changes to species distributions under climate change. Climate change will likely affect species future distributions and the optimization of protected areas is a necessity in global change scenarios. Reaching the decision makers with this information is therefore of the upmost importance.

## Supporting information

S1 Fig Map of the current protected areas for the Islands of Terceira and São Miguel in the Azores. Different colors represent the different levels of protection according to the IUCN (data provided by the Regional Directorate of the Environment–public record).
(TIF)

S2 Fig Projections for locations of protected areas calculated with the PRION software for São Miguel Island for the 1961–90 and 2080–99 periods for Bryophytes, and the current protected areas. Minimum Set—quasi-optimal solution that minimized the protected number of cells while protecting the same average proportion of each species’ range. Maximum Coverage—the optimal solution that maximized species coverage with similar costs.
(TIF)

S3 Fig Projections for locations of protected areas calculated with the PRION software for São Miguel Island for the 1961–90 and 2080–99 periods for Vascular plants, and the current protected areas. Minimum Set—quasi-optimal solution that minimized the protected number of cells while protecting the same average proportion of each species’ range. Maximum Coverage—the optimal solution that maximized species coverage with similar costs.
(TIF)

S4 Fig Projections for locations of protected areas calculated with the PRION software for São Miguel Island for the 1961–90 and 2080–99 periods for Arthropods, and the current protected areas. Minimum Set—quasi-optimal solution that minimized the protected number of cells while protecting the same average proportion of each species’ range. Maximum Coverage—the optimal solution that maximized species coverage with similar costs.
(TIF)

S5 Fig Projections for locations of protected areas calculated with the PRION software for Terceira Island for the 1961–90 and 2080–99 periods for Bryophytes, and the current protected areas. Minimum Set—quasi-optimal solution that minimized the protected number of cells while protecting the same average proportion of each species’ range. Maximum Coverage—the optimal solution that maximized species coverage with similar costs.
(TIF)

S6 Fig Projections for locations of protected areas calculated with the PRION software for Terceira Island for the 1961–90 and 2080–99 periods for Vascular plants, and the current protected areas. Minimum Set—quasi-optimal solution that minimized the protected number of cells while protecting the same average proportion of each species’ range. Maximum Coverage—the optimal solution that maximized species coverage with similar costs.
(TIF)

S7 Fig Projections for locations of protected areas calculated with the PRION software for Terceira Island for the 1961–90 and 2080–99 periods for Vascular plants, and the current protected areas. Minimum Set—quasi-optimal solution that minimized the protected number of cells while protecting the same average proportion of each species’ range. Maximum Coverage—the optimal solution that maximized species coverage with similar costs.
(TIF)

S8 Fig Land use and current protected areas for São Miguel Island (data provided by the Regional directorate for the territorial planning and hydric resources–public record). Different colors represent the different types of land use.
(TIF)

S9 Fig Land use and current protected areas for Terceira Island (data provided by the Regional directorate for the territorial planning and hydric resources–public record). Different colors represent the different types of land use.
(TIF)

S1 Table List of Azorean endemic species used for the analysis. Levels of protection and Island distribution are provided.
(PDF)

S2 Table List of variables used for the CIELO model. Acronyms and full description of variables are provided.
(PDF)

S3 Table List of methods used in the BioEnsembles software. Methods and references are provided.
(PDF)

S4 Table Null model analysis for each taxonomic group and for the complete set of species for both Terceira and São Miguel Islands. Number and percentage of species that are equal, higher or less than the null model are presented.
(PDF)
