# Correction: Implications of climate change to the design of protected areas: The case study of small islands (Azores)

**DOI:** 10.1371/journal.pone.0219583

**Published:** 2019-07-10

**Authors:** Maria Teresa Ferreira, Pedro Cardoso, Paulo A. V. Borges, Rosalina Gabriel, Eduardo Brito de Azevedo, Rui Bento Elias

The following information is missing from the Funding section: Open access was partly financed by Direção Regional da Ciência e Tecnologia—DRCT (DRCT/2019/M3.3.c/001) and Fundação para a Ciência e a Tecnologia—FCT (UID/BIA/00329/2019) funds. The funders had no role in study design, data collection and analysis, decision to publish, or preparation of the manuscript.
