# Supplementary figures and images for: Implications of climate change to the design of protected areas: The case study of small islands (Azores)
Source: PLoS One. 2019 Jun 13;14(6):e0218168. doi: 10.1371/journal.pone.0218168 (PMC6563998; doi:10.1371/journal.pone.0218168)

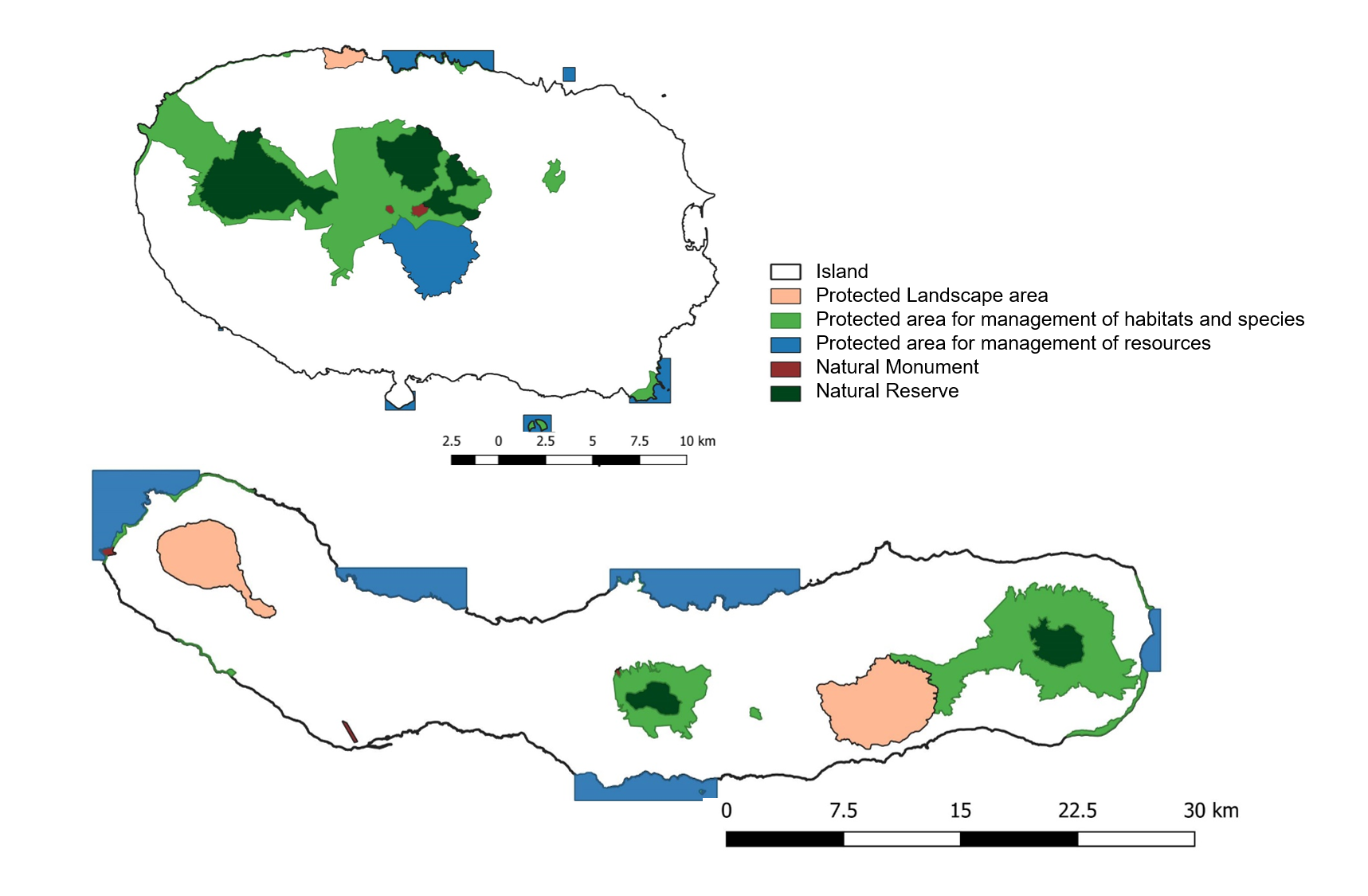

Supplement: S1 Fig — Different colors represent the different levels of protection according to the IUCN (data provided by the Regional Directorate of the Environment–public record). (TIF) [file pone.0218168.s001.tif]

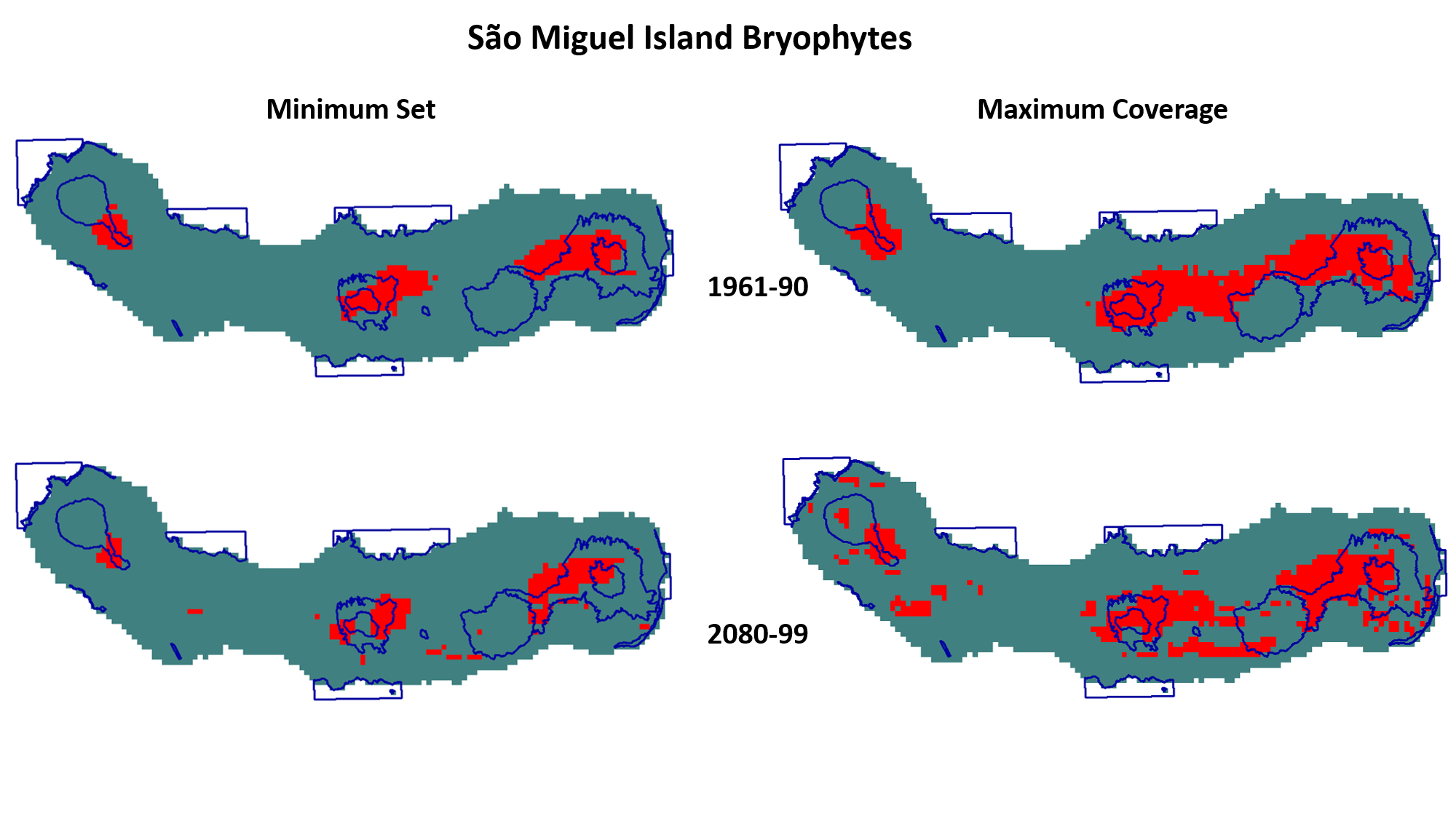

Supplement: S2 Fig — Minimum Set—quasi-optimal solution that minimized the protected number of cells while protecting the same average proportion of each species’ range. Maximum Coverage—the optimal solution that maximized species coverage with similar costs. (TIF) [file pone.0218168.s002.tif]

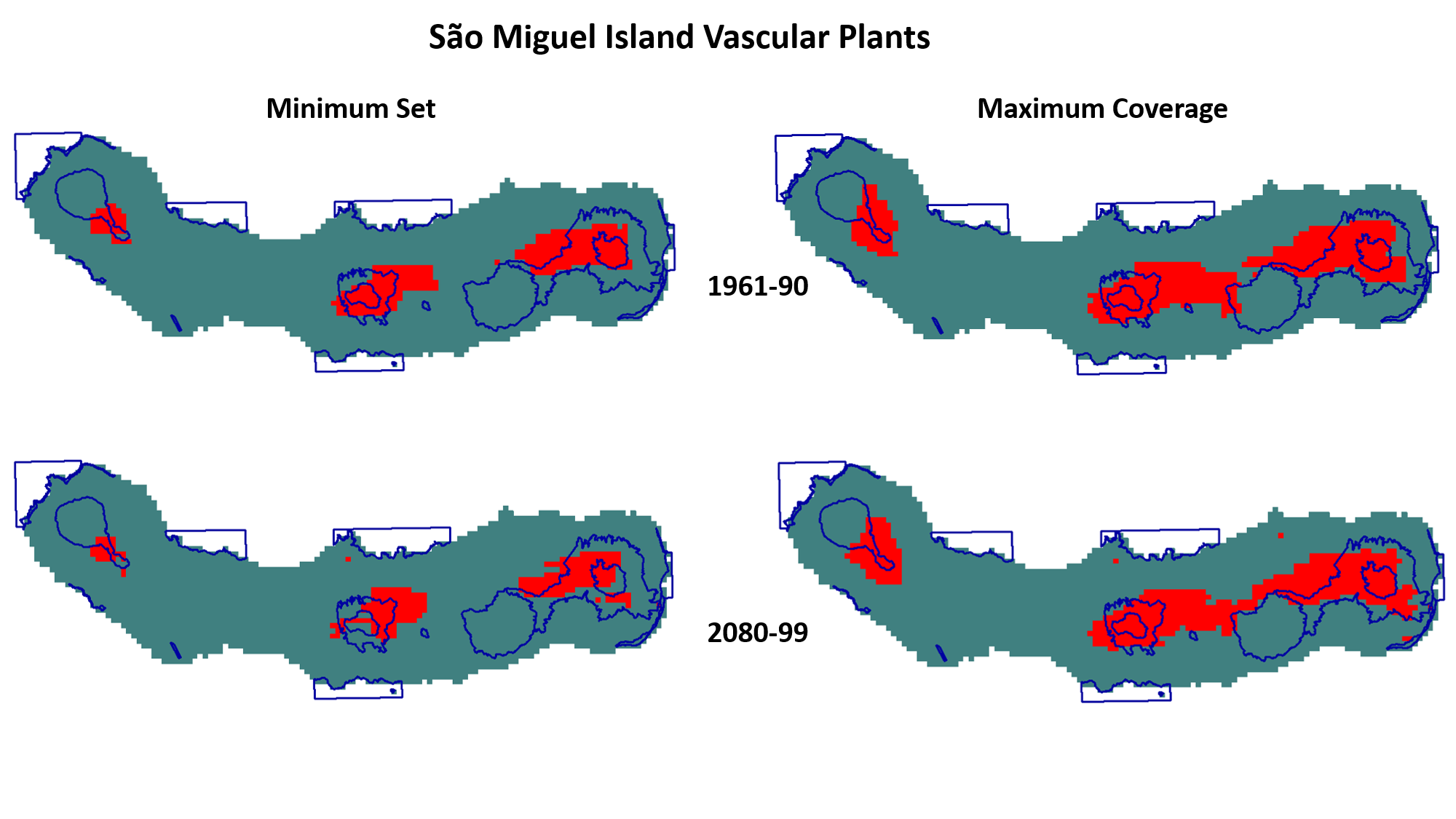

Supplement: S3 Fig — Minimum Set—quasi-optimal solution that minimized the protected number of cells while protecting the same average proportion of each species’ range. Maximum Coverage—the optimal solution that maximized species coverage with similar costs. (TIF) [file pone.0218168.s003.tif]

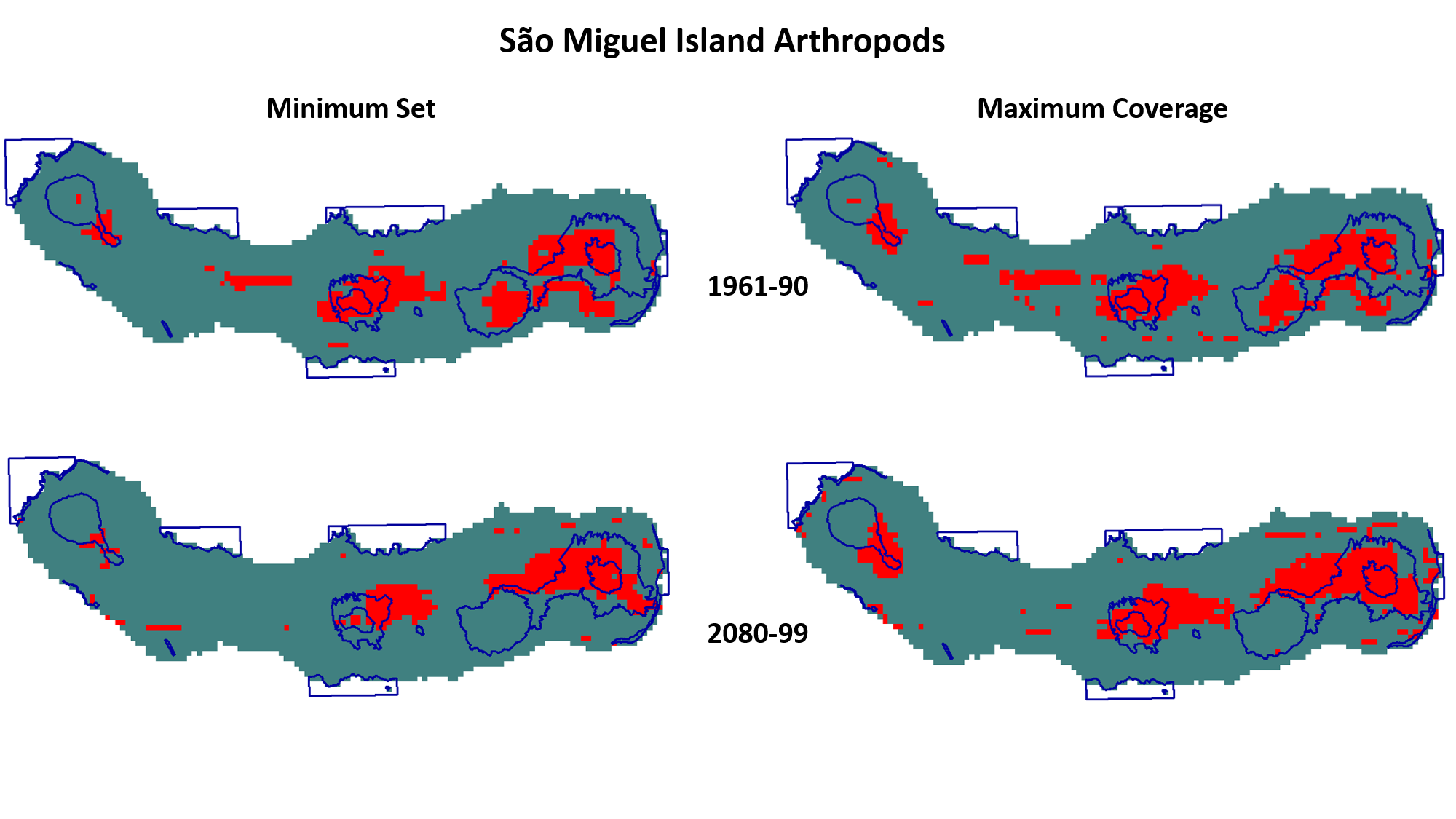

Supplement: S4 Fig — Minimum Set—quasi-optimal solution that minimized the protected number of cells while protecting the same average proportion of each species’ range. Maximum Coverage—the optimal solution that maximized species coverage with similar costs. (TIF) [file pone.0218168.s004.tif]

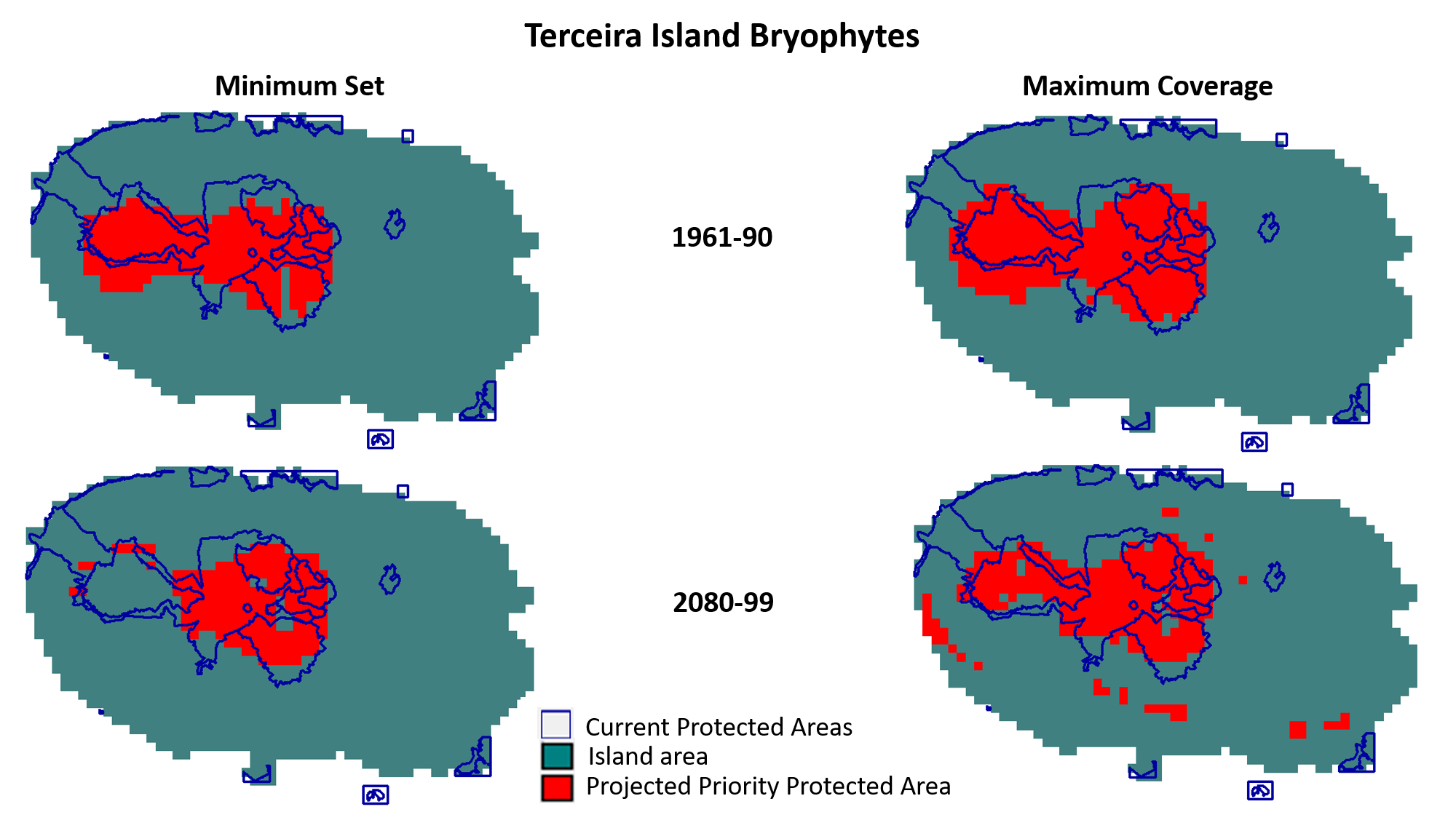

Supplement: S5 Fig — Minimum Set—quasi-optimal solution that minimized the protected number of cells while protecting the same average proportion of each species’ range. Maximum Coverage—the optimal solution that maximized species coverage with similar costs. (TIF) [file pone.0218168.s005.tif]

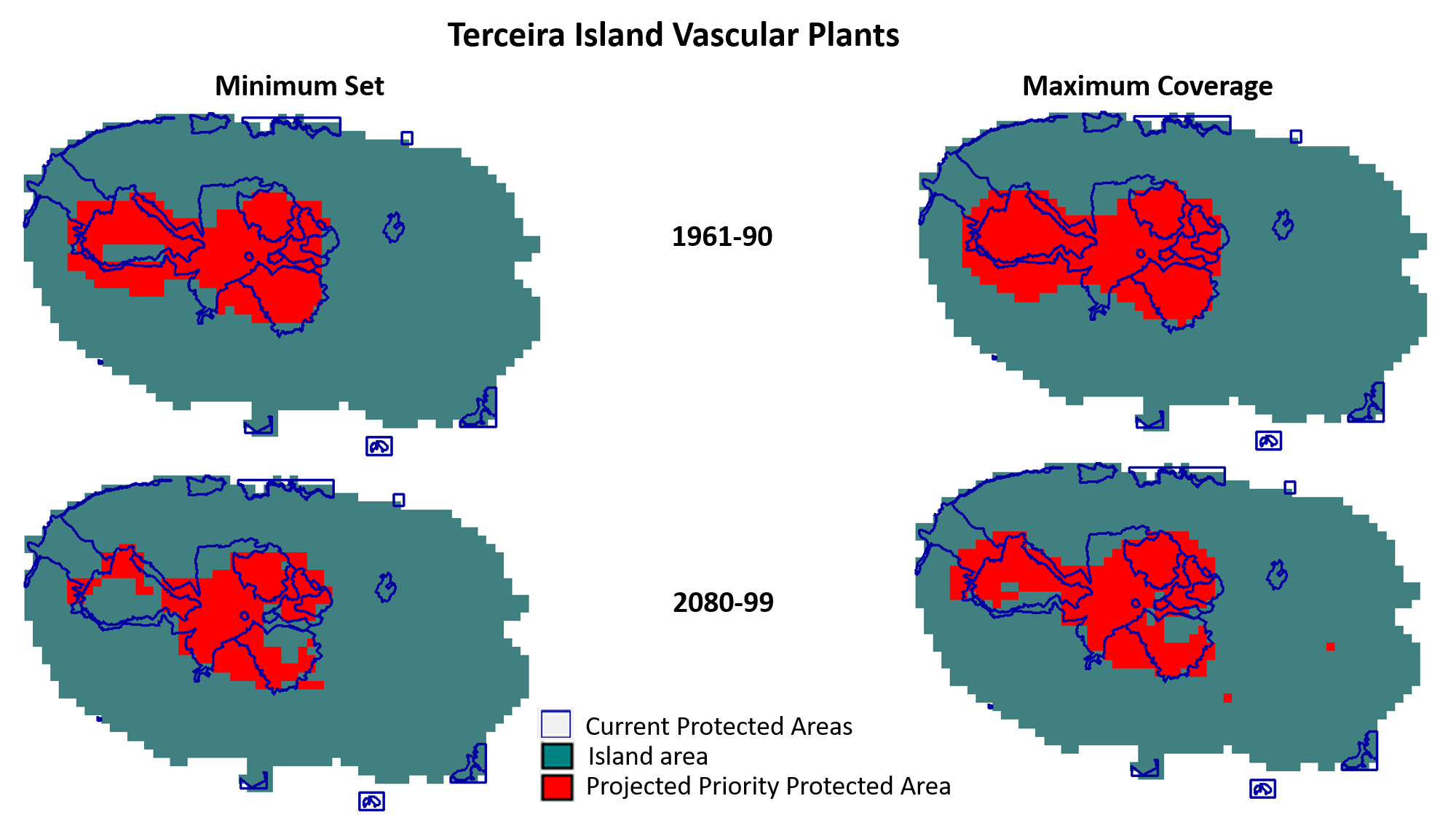

Supplement: S6 Fig — Minimum Set—quasi-optimal solution that minimized the protected number of cells while protecting the same average proportion of each species’ range. Maximum Coverage—the optimal solution that maximized species coverage with similar costs. (TIF) [file pone.0218168.s006.tif]

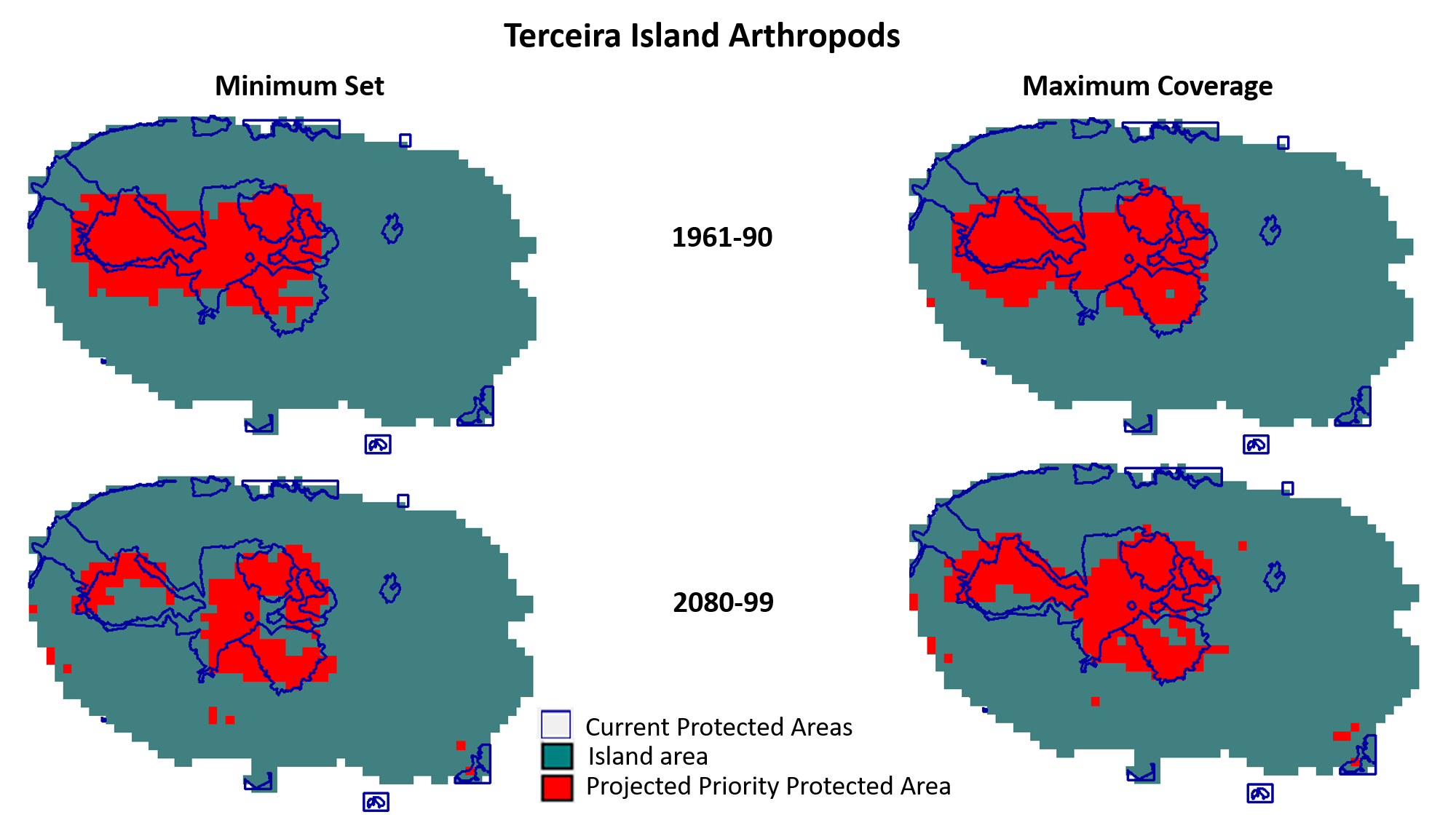

Supplement: S7 Fig — Minimum Set—quasi-optimal solution that minimized the protected number of cells while protecting the same average proportion of each species’ range. Maximum Coverage—the optimal solution that maximized species coverage with similar costs. (TIF) [file pone.0218168.s007.tif]

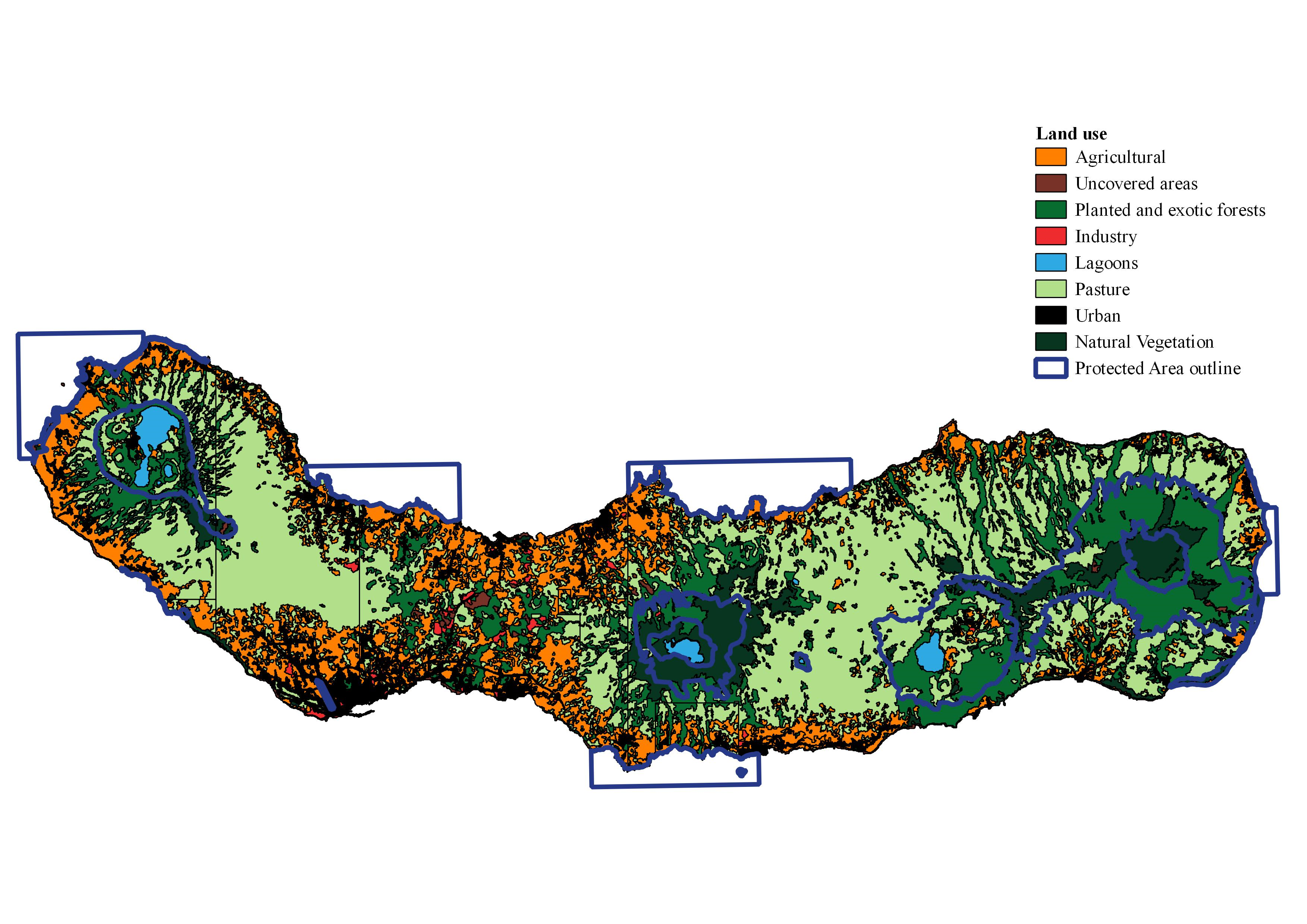

Supplement: S8 Fig — Different colors represent the different types of land use. (TIF) [file pone.0218168.s008.tif]

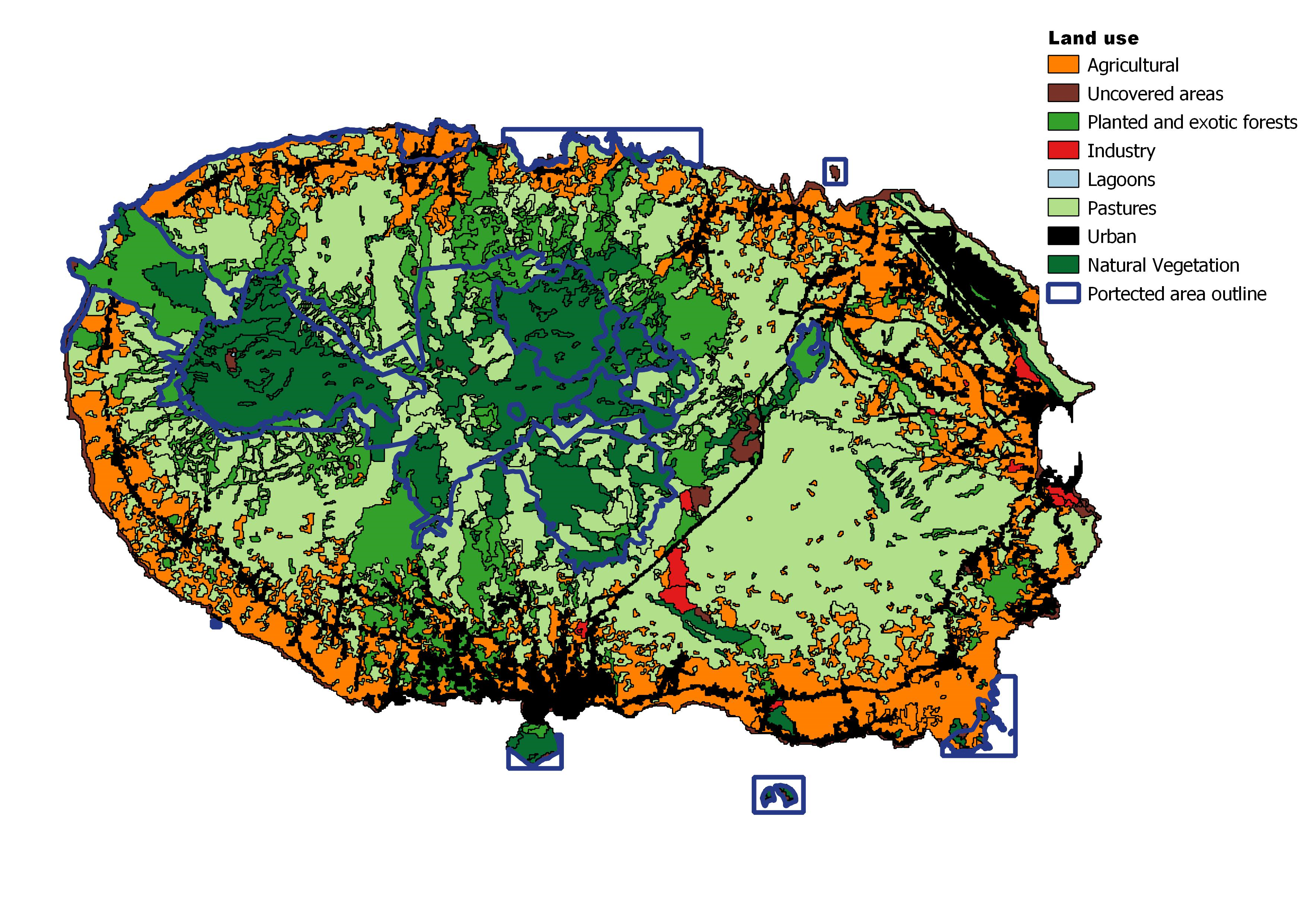

Supplement: S9 Fig — Different colors represent the different types of land use. (TIF) [file pone.0218168.s009.tif]
