# Supplementary material for: Implications of climate change to the design of protected areas: The case study of small islands (Azores)
Source: PLoS One. 2019 Jun 13;14(6):e0218168. doi: 10.1371/journal.pone.0218168 (PMC6563998; doi:10.1371/journal.pone.0218168)
Supplement: S1 Table — Levels of protection and Island distribution are provided. (PDF) [file pone.0218168.s010.pdf]

S1 Table. List of endemic Azorean species used, their IUCN status and Islands where they are present

Taxonomic group	Species name	IUCN	Islands
Bryophytes	*Bazzania azorica*	NE	FLO, FAI, PIC, SJG, TER, SMG
	Breutelia azorica	NE	FLO, FAI, PIC, SJG, TER, SMG
	Echinodium renauldii	VU	COR, FLO, FAI, PIC, SJG, TER, SMG
	Leptoscyphus porfirius subsp. *azoricus*	NE	FLO, PIC, SJG, TER, SMG
	Sphagnum nitidulum	NE	TER
	Thamnobryum rudolphianum	NE	COR, FAI, PIC, SJG, TER
	Trematodon perssoniorum	NE	SMG
Vascular Plants	*Agrostis congestiflora congestiflora*	NE	COR, FLO, FAI, PIC, GRA, SJG, TER, SMG
	Agrostis gracililaxa	NE	FLO, FAI, PIC, GRA, TER, SMG
	Ammi seubertianum	NE	PIC, SMG, SMR
	Angelica lignescens	NE	FLO, FAI, PIC, TER, SMG
	Arceuthobium azoricum	NE	FAI, PIC, SJG, TER, SMG
	Asplenium azoricum	LC	COR, FLO, FAI, PIC, GRA, SJG, TER, SMG, SMR
	Azorina vidalii	EN	COR, FLO, FAI, PIC, GRA, SJG, TER, SMG, SMR
	Cardamine caldeirarum	NE	COR, FLO, FAI, PIC, SJG, TER, SMG, SMR
	Carex hochstetteriana	NE	COR, FLO, FAI, PIC, SJG, TER, SMG, SMR
	Carex pilulifera azorica	NE	FLO, FAI, PIC, SJG, TER, SMG
	Carex vulcani	NE	FLO, FAI, PIC, SJG, TER, SMG, SMR
	Daucus carota azorica	NE	COR, FLO, FAI, PIC, GRA, SJG, TER, SMG, SMR
	Deschampsia foliosa	NE	COR, FLO, FAI, PIC, SJG, TER, SMG
	Dryopteris azorica	NE	COR, FLO, FAI, PIC, GRA, SJG, TER, SMG, SMR
	Dryopteris crispifolia	LC	COR, FLO, FAI, PIC, TER, SMG
	Erica azorica	NE	COR, FLO, FAI, PIC, GRA, SJG, TER, SMG, SMR
	Euphorbia azorica	NE	COR, FLO, FAI, PIC, GRA, SJG, TER, SMG, SMR
	Euphorbia stygiana stygiana	NE	COR, FLO, FAI, PIC, SJG, TER, SMG
	Euphrasia grandiflora	NE	PIC, SJG, TER
	Festuca francoi	NE	COR, FLO, FAI, PIC, GRA, SJG, TER, SMG, SMR
	Festuca petraea	NE	COR, FLO, FAI, PIC, GRA, SJG, TER, SMG, SMR
	Frangula azorica	LC	FLO, FAI, PIC, SJG,TER, SMG

Vascular Plants	*Gaudinia coarctata*	NE	COR, FLO, FAI, PIC, GRA, SJG, TER, SMG, SMR
	Hedera azorica	NE	COR, FLO, FAI, PIC, GRA, SJG, TER, SMG, SMR
	Holcus azoricus	NE	FAI, PIC, GRA, SJG, TER, SMG, SMR
	Holcus rigidus	NE	COR, FLO, FAI, PIC, GRA, SJG, TER, SMG, SMR
	Hypericum foliosum	LC	COR, FLO, FAI, PIC, GRA, SJG, TER, SMG, SMR
	Ilex perado azorica	NE	COR, FLO, FAI, PIC, GRA, SJG, TER, SMG, SMR
	Isoetes azorica	VU	COR, FLO, FAI, PIC, SJG, TER
	Juniperus brevifolia	VU	COR, FLO, FAI, PIC, SJG, TER, SMG, SMR
	Lactuca watsoniana	EN	FAI, PIC, SJG, TER, SMG
	Laurus azorica	LC	COR, FLO, FAI, PIC, GRA, SJG, TER, SMG, SMR
	Leontodon filii	NE	PIC, SJG, TER, SMG
	Leontodon rigens	NE	PIC, TER, SMG
	Luzula purpureosplendens	NE	COR, FLO, FAI, PIC, SJG, TER, SMG
	Lysimachia azorica	NE	COR, FLO, FAI, PIC, GRA, SJG, TER, SMG, SMR
	Myosotis maritima	NE	COR, FLO, FAI, PIC, GRA, SJG, TER, SMR
	Myrsine africana	NE	COR, FLO, FAI, PIC, GRA, SJG, TER, SMG, SMR
	Pericallis malvifolia malvifolia	NE	FAI, PIC, SJG, TER, SMG, SMR
	Picconia azorica	LC	COR, FLO, FAI, PIC, SJG, TER, SMG, SMR
	Platanthera micrantha	EN	COR, FLO, FAI, PIC, SJG, TER, SMG, SMR
	Polypodium azoricum	NE	COR, FLO, FAI, PIC, GRA, SJG, TER, SMG, SMR
	Prunus azorica	NE	FLO, FAI, PIC, SJG, TER, SMG
	Rubus hochstetterorum	LC	COR, FLO, FAI, PIC, SJG, TER, SMG, SMR
	Rumex azoricus	NE	COR, FAI, SJG, TER, SMG
	Smilax azorica	NE	FAI, PIC, GRA, SJG, TER, SMG, SMR
	Spergularia azorica	NE	COR, FLO, FAI, PIC, GRA, SJG, TER, SMG, SMR
	Tolpis azorica	NE	COR, FLO, FAI, PIC, SJG, TER, SMG
	Vaccinium cylindraceum	LC	COR, FLO, FAI, PIC, SJG, TER, SMG, SMR
	Viburnum treleasei	LC	COR, FLO, FAI, PIC, SJG, TER, SMG, SMR
**Arthropods**	*Acorigone acoreensis*	NE	FLO, FAI, PIC, SJG, TER, SMG, SMR
	Agabus godmani	NE	FLO, FAI, PIC, GRA, SJG, TER, SMG
	Agyneta rugosa	NE	FAI, SJG, SMG

**Arthropods**	*Alestrus dolosus*	DD	FLO, FAI, PIC, TER, SMG, SMR
	Aphaniosoma azoricum	NE	FLO, FAI, SJG, TER, SMG
	Aphrodes hamiltoni	EN	FLO, FAI, PIC, GRA, SJG, TER, SMG, SMR
	Aphrosylus argyreatus	NE	COR, FLO, FAI, TER, SMG
	Aphrosylus calcarator	NE	COR, FLO, FAI, PIC, GRA, SJG, TER, SMG
	Argyresthia atlanticella	NT	COR, FLO, FAI, PIC, GRA, SJG, TER, SMG, SMR
	Argyresthia minusculella	EN	FLO, PIC, TER
	Ascotis fortunata azorica	NE	COR, FLO, FAI, PIC, GRA, SJG, TER, SMG, SMR
	Atheta caprariensis	CR	SMG
	Atheta dryochares	NT	FAI, PIC, GRA, SJG, TER, SMG, SMR
	Athous azoricus	EN	GRA, TER, SMG
	Atlantocis gillerforsi	EN	FLO, PIC, TER, SMG, SMR
	Azorastia minutissima	NE	FLO, SJG, SMG
	Bembidion schmidti mequignoni	NE	FLO, FAI, PIC, SMG
	Calacalles subcarinatus	LC	COR, FLO, FAI, PIC, GRA, SJG, TER, SMG, SMR
	Calathus lundbladi	CR	SMG
	Campsicnemus mirabilis	EX	TER, SMG
	Canariphantes acoreensis	NE	FAI, PIC, SJG, TER
	Cedrorum azoricus azoricus	NE	TER, SMR
	Cerodontha bistrigata	NE	COR, FAI, PIC, SJG, TER
	Chaetophiloscia guernei	NE	COR, FLO, FAI, PIC, GRA, TER, SMG, SMR
	Chrysotus elongatus	NE	COR, FLO, FAI, PIC, SJG, TER, SMG
	Chrysotus vulcanicola	NE	FLO, FAI, PIC, SJG, TER, SMG
	Cixius azoricus azoricus	NE	FAI, SJG, TER, SMG
	Cixius azoterceirae	VU	TER
	Cixius insularis	VU	SMG
	Coenosia freyi semicandida	NE	SMG
	Coenosia testacea azorica	NE	FAI, PIC, TER, SMG
	Conocephalus chavesi	EN	PIC, TER, SMG
	Cyclophora azorensis	LC	COR, FLO, FAI, PIC, GRA, SJG, TER, SMG, SMR
	Damaeus pomboi	NE	SJG, TER, SMG, SMR

Arthropods	*Dicranomyia azorica*	NE	FAI, SMG
	Discobola freyana	NE	FLO, FAI, SMG
	Dolichopus anacrostichus	NE	SJG, TER, SMG
	Drouetius borgesi borgesi	NE	TER
	Drouetius oceanicus oceanicus	NE	TER
	Elipsocus azoricus	LC	COR, FLO, FAI, PIC, GRA, SJG, TER, SMG, SMR
	Elipsocus brincki	LC	COR, FLO, FAI, PIC, GRA, SJG, TER, SMG, SMR
	Emblyna acoreensis	NE	COR, FLO, FAI, PIC, GRA, SJG, TER
	Encarsia estrellae	NE	PIC, SMG
	Ensina azorica	NE	COR, FLO, PIC, GRA, SJG, TER, SMG
	Euconnus azoricus	EN	PIC, TER, SMG
	Eudarcia atlantica	EN	FAI, TER, SMG
	Eudonia interlinealis	LC	COR, FLO, FAI, PIC, GRA, SJG, TER, SMG, SMR
	Eudonia luteusalis	LC	FLO, FAI, PIC, SJG, TER, SMG, SMR
	Eudonia melanographa	VU	FLO, FAI, PIC, SJG, TER, SMG
	Eupteryx azorica	NT	COR, FLO, PIC, GRA, SJG, TER, SMG
	Gibbaranea occidentalis	NE	FLO, FAI, PIC, GRA, SJG, TER, SMG, SMR
	Graphania granti	EN	PIC, TER, SMG
	Hemerobius azoricus	LC	FLO, FAI, PIC, GRA, SJG, TER, SMG, SMR
	Heminothrus oromii	NE	TER, SMG
	Heteroderes azoricus	EN	COR, FLO, FAI, PIC, GRA, SJG, TER, SMG, SMR
	Hipparchia azorina azorina	NE	FAI, PIC, SJG, TER
	Hipparchia miguelensis	LC	SMG
	Homoeosoma miguelensis	CR	SMG
	Hydroporus guernei	EN	COR, FLO, FAI, PIC, SJG, TER, SMG, SMR
	Jaera nordmanni	NE	COR, FLO, FAI, PIC, GRA, SJG, TER, SMG, SMR
	Javesella azorica	NT	COR, FLO, FAI, PIC, SJG, TER, SMG
	Kowarzia dahli	NE	FAI, SMG
	Kowarzia sexmaculata	NE	SJG, TER
	Lasaeola oceanica	NE	COR, FLO, FAI, PIC, GRA, SJG, TER, SMG, SMR
	Liacarus angustatus	NE	FAI, PIC, TER, SMG, SMR

**Arthropods**	*Limnellia helmuti*	NE	SMG
	Limnephilus atlanticus	NT	COR, FLO,FAI,PIC, SJG, TER, SMG
	Lithobius obscurus azoreae	NE	FAI, PIC, GRA, TER
	Melanozetes azoricus sanctaemariae	NE	TER, SMG, SMR
	Mesapamea storai	LC	COR, FLO, FAI, PIC, GRA, SJG, TER, SMG
	Minicia floresensis	NE	FLO, PIC, SJG, TER, SMG
	Neomariania oecophorella	VU	FLO, FAI, PIC, TER, SMG
	Neon acoreensis	NE	FLO, FAI, PIC, SJG, TER, SMG, SMR
	Nesotes azoricus	CR	SMG
	Noctua atlantica	LC	COR, FLO, FAI, PIC, GRA, SJG, TER, SMG
	Nothrus palustris azorensis	NE	FLO, GRA, TER, SMG, SMR
	Nysius atlantidum	LC	FLO, FAI, GRA, TER, SMG, SMR
	Orchestia chevreuxi	NE	COR, FLO, FAI, PIC, GRA, TER, SMG, SMR
	Pardosa acorensis	NE	COR, FLO, FAI, PIC, GRA, SJG, TER, SMG, SMR
	Philygria cedercreutzi	NE	FLO, TER
	Phloeosinus gillerforsi	EN	FLO, PIC, SJG, TER, SMG
	Phloeostiba azorica	EN	FLO, PIC, GRA, SJG, TER, SMG
	Phlogophora cabrali	NT	FAI, PIC, SJG, SMG
	Phlogophora furnasi	VU	PIC, SJG, TER, SMG
	Phlogophora interrupta	LC	COR, FLO, FAI, PIC, GRA, SJG, TER, SMG, SMR
	Pieris brassicae azorensis	NE	COR, FLO, FAI, PIC, GRA, SJG, TER, SMG, SMR
	Pinalitus oromii	LC	FLO, FAI, PIC, GRA, SJG, TER, SMG, SMR
	Pisaura acoreensis	NE	FLO, FAI, PIC, GRA, SJG, TER, SMG, SMR
	Polydesmus ribeiraensis	NE	SMG
	Porrhomma borgesi	NE	PIC, TER, SMG
	Pseudanchomenus aptinoides	CR	PIC, SMG
	Pseudechinosoma nodosum	EN	FLO, FAI, PIC, TER, SMG, SMR
	Pseudolycoriella campanulata	NE	FLO, TER, SMG
	Pseudosinella ashmoleorum	NE	FAI, PIC, TER
	Rachispoda atrolimosa	NE	FLO, SJG, TER, SMG
	Rugathodes acoreensis	NE	FLO, FAI, PIC, GRA, SJG, TER, SMG, SMR

**Arthropods**	*Rymosia azorensis*	NE	SMG
	Sancus acoreensis	NE	FLO, FAI, PIC, SJG, TER, SMG, SMR
	Savigniorrhipis acoreensis	NE	FLO, FAI, PIC, SJG, TER, SMG, SMR
	Scaptomyza impunctata	NE	FLO, FAI, PIC, SJG, TER, SMG
	Sciapus glaucescens brioni	NE	FAI, PIC, SMG
	Scoparia aequipennalis	LC	COR, FLO, FAI, PIC, GRA, SJG, TER, SMG, SMR
	Scoparia carvalhoi	VU	FAI, PIC, TER, SMR
	Scoparia semiamplalis	NE	FLO, FAI, PIC, SJG, TER, SMG, SMR
	Sepsis nephodes	NE	TER
	Simulium azorense	NE	FLO, FAI, SJG, TER, SMG, SMR
	Sphaerophoria nigra	NE	FLO, FAI, PIC, SJG, TER, SMG
	Sphaerophoria philanthus	NE	SMG
	Steganacarus hirsutus azorensis	NE	GRA, TER, SMG, SMR
	Strophingia harteni	LC	COR, FLO, FAI, PIC, GRA, SJG, TER, SMG, SMR
	Tarphius azoricus	EN	FLO, SMG
	Tarphius depressus	CR	PIC, SMG, SMR
	Tarphius tornvalli	EN	SMG
	Tinea poecilella	CR	SMG
	Trechus terceiranus	VU	TER
	Trechus terrabravensis	EN	TER
	Trigoniophthalmus borgesi	EN	FAI, PIC, SJG, TER, SMG, SMR
	Trixoscelis proxima	NE	SMG
	Udea azorensis	NT	FLO, PIC, SJG, TER, SMG
	Walckenaeria grandis	NE	FLO, PIC, SJG, TER, SMG
	Xanthandrus azorensis	NE	FAI, PIC, SJG, SMG
	Xanthorhoe inaequata	LC	COR, FLO, FAI, PIC, GRA, SJG, TER, SMG, SMR

IUCN protection status: NE - Not evaluated; DD - Data Deficient; LC- Least Concern; NT - Near Threathened; VU - Vulnerable; EN - Endangered; CR - Critically Endangered; EW - Extinct in the wild; EX - extinct

Islands of the Azores: COR- Corvo; FLO - Flores; FAI - Faial; PIC - Pico; GRA - Graciosa; SJG - São Jorge; TER - Terceira; SMG - São Miguel; SMR - Santa Maria.
