# Supplementary material for: Implications of climate change to the design of protected areas: The case study of small islands (Azores)
Source: PLoS One. 2019 Jun 13;14(6):e0218168. doi: 10.1371/journal.pone.0218168 (PMC6563998; doi:10.1371/journal.pone.0218168)
Supplement: S2 Table — Acronyms and full description of variables are provided. (PDF) [file pone.0218168.s011.pdf]

S2 Table. List of variables used for the CIELO model.

Variables	Acronym
maximum annual temperature	tmax
minimum annual temperature	tmin
annual range of temperature	trange
average temperature	tmed
maximum annual precipitation	ppmax
mimnimum annual precipitation	ppmin
annual range of precipitation	prange
average precipitation	ppmed
maximum annual relative humidity	rhmax
minimum annual relative humidity	rhmin
annual range of relative humidity	rhrange
average annual relative humidity	rhmed
solar radiation	radsol

All values were obtained from local long term weather stations
