# Supplementary material for: Implications of climate change to the design of protected areas: The case study of small islands (Azores)
Source: PLoS One. 2019 Jun 13;14(6):e0218168. doi: 10.1371/journal.pone.0218168 (PMC6563998; doi:10.1371/journal.pone.0218168)
Supplement: S3 Table — Methods and references are provided. (PDF) [file pone.0218168.s012.pdf]

S3 Table- Models used for the BIOENSEMBLES software

Models used	References
Mahalanobis distance (MD)	Mahalanobis 1936
Ecological Niche Factor Analysis (ENFA)	Hirzel et al. 2002
BIOCLIM	Kriticos et al. 2012
Maximum Entropy (MaxEnt)	Phillips et al. 2006
Genetic Algorithm for Rule Prediction (GARP)	Stockwell and Peters 1999
Random Forests (RF)	Breiman 2001
Multiple Additive Regression Splines (MARS)	Friedman 1991
Flexible Discriminant Analysis (FDA)	Hastie et al. 1993
Euclidean distance (ED)	
Gower distance (GD)	
Generalised Linear Models (GLM)	
Generalised Additive Models (GAM)	
Generalized Boosting Models (GMB)	
Artificial Neural Networks (ANN)	
