# Supplementary material for: Implications of climate change to the design of protected areas: The case study of small islands (Azores)
Source: PLoS One. 2019 Jun 13;14(6):e0218168. doi: 10.1371/journal.pone.0218168 (PMC6563998; doi:10.1371/journal.pone.0218168)
Supplement: S4 Table — Number and percentage of species that are equal, higher or less than the null model are presented. (PDF) [file pone.0218168.s013.pdf]

S4 Table. Null model analysis for each taxonomic group and for the complete set of species for both Terceira and São Miguel Islands.

Taxonomic group	Island	Time period	Current Protected Areas			Minimum Set			Maximum Coverage			
			> expected	= expected	< expected	> expected	= expected	< expected	> expected	= expected	< expected	Total
All taxonomic groups	São Miguel	1961-90	139 (90%)	7 (5%)	8 (5%)	101 (66%)	0 (0%)	53 (34%)	101 (66%)	4 (2%)	49 (32%)	154
		2080-99	133 (89%)	14 (9%)	2 (1%)	106 (71%)	13 (9%)	30 (20%)	111 (74%)	18 (12%)	20 (13%)	149
	Terceira	1961-90	105 (71%)	18 (12%)	25 (17%)	100 (68%)	4 (2%)	44 (30%)	99 (67%)	5 (3%)	44 (30%)	148
		2080-99	105 (79%)	8 (6%)	20 (15%)	105 (79%)	9 (7%)	19 (14%)	107 (80%)	6 (5%)	20 (15%)	133
Bryophytes	São Miguel	1961-90	5 (100%)	0 (0%)	0 (0%)	3 (60%)	0 (0%)	2 (40%)	3 (60%)	0 (0%)	2 (40%)	5
		2080-99	5 (100%)	0 (0%)	0 (0%)	4 (80%)	1 (20%)	0 (0%)	5 (100%)	0 (0%)	0 (0%)	5
	Terceira	1961-90	5 (83%)	1 (17%)	0 (0%)	5 (83%)	0 (0%)	1 (17%)	5 (83%)	0 (0%)	1 (17%)	6
		2080-99	5(83%)	0 (0%)	1 (13%)	5 (83%)	0 (0%)	1 (17%)	6 (100%)	0 (0%)	0 (0%)	6
Vascular Plants	São Miguel	1961-90	40 (87%)	3 (7%)	3 (7%)	36 (78%)	0 (0%)	10 (22%)	36 (78%)	0 (0%)	10 (22%)	46
		2080-99	44 (92%)	3 (6%)	1 (2%)	41 (85%)	2 (4%)	5 (11%)	40 (83%)	2 (4%)	6 (13%)	48
	Terceira	1961-90	36 (76%)	6 (12%)	6 (12%)	36 (73%)	1 (2%)	12 (24%)	37 (72%)	0 (0%)	12 (24%)	49
		2080-99	36 (82%)	1 (2%)	7 (16%)	36 (82%)	1 (2%)	7 (16%)	37 (84%)	0 (0%)	7 (16%)	44
Arthropods	São Miguel	1961-90	94 (91%)	4 (4%)	5 (5%)	98 (95%)	4 (4%)	1 (1%)	98 (95%)	0 (0%)	5 (5%)	103
		2080-99	89 (90%)	9 (9%)	1 (1%)	82 (83%)	9 (9%)	8 (8%)	86 (87%)	7 (7%)	6 (6%)	99
	Terceira	1961-90	63 (68%)	11 (12%)	19 (20%)	58 (62%)	5 (5%)	30 (32%)	59 (63%)	4 (4%)	30 (32%)	93
		2080-99	63 (76%)	7 (8%)	13 (16%)	66 (80%)	9 (11%)	8 (10%)	66 (80%)	6 (7%)	11 (13%)	83

In both periods: the current PA network; the quasi-optimal solution that minimized the protected number of cells while protecting the same average proportion of each species' range ('minimum set'); and the optimal solution that maximized species coverage with similar costs ('maximum coverage'). > null is number and percentage of species with values better than what could be expected by random placement of protected cells; <null is number and percentage of species with values worse than what could be expected by random placement of protected cells; and = null is number and percentage of species with values not different from what could be expected by random placement of protected cells. Note: Although current PAs seem to have a better performance, they only aim to optimize protection for a few species, based on which the areas were created, while the optimization techniques are optimizing for all the species both inside and outside the PA.
